# Anti-biofouling adsorptive sheet based on polyethersulfone/ dried algal biomass / ZnO nanoparticles for dyes removal

**DOI:** 10.1038/s41598-025-02081-0

**Published:** 2025-05-21

**Authors:** Doaa Hussein, Ahmed E. Abdelhamid, Mohamed Gad, Einas El-shatoury, Ahmed Labena

**Affiliations:** 1 National Institute of Occupational Safety and Health (NIOSH), Misr Elgadida, Cairo, Egypt; 2 Polymers and Pigments Department, National Research Centre, Dokki, 12622 Cairo Egypt; 3 Microbiology Department, Faculty of Science, Ain Shams University, Cairo, Egypt; 4 Egyptian Petroleum Research Institute (EPRI), Nasr City, 11727 Cairo Egypt

**Keywords:** Anti-biofouling, Polymer composite, Wastewater treatment, Crystal Violet, Methylene blue, *Sargassum dentifolium*, Environmental sciences, Chemistry

## Abstract

This study aims to develop an environmentally friendly composite matrix for removing dyes from wastewater. The composite matrix was prepared by incorporating finely ground biomass of the seaweed *Sargassum dentifolium* (S) and zinc oxide nanoparticles (ZnO) into polyethersulfone (PES) forming composite sheets (PES-S-ZnO). Composite sheets were characterized by Attenuated Total Reflectance Fourier Transform Infrared (ATR)-FTIR, Scanning Electron Microscopy (SEM), and Energy dispersive X-ray (EDX), as well as the swelling behavior, porosity & leaching of ZnO nanoparticles from the sheets were determined. The composite sheet with 20% *Sargassum*, has proven to be the most effective dye bio-sorbent. Crystal violet (CV), Methylene blue (MB) and Congo red (CR) were successfully removed from the contaminated waters within 6, 6 and 12 h, with removal efficiency of 92.46, 93.10 and 37.96%, respectively. Langmuir isotherm and pseudo-second order kinetic studies could explain the composite bio-sorbent behavior. Furthermore, the recovery and reuse results confirmed that the polymer sheets have stable performance after 5 cycles. The accelerated weathering for the composite sheets indicated that the incorporation of *Sargassum* biomass into the sheets increased the probability of sheets’ degradation. Furthermore, increasing the ZnO nanoparticles in sheets leads to an increase in anti-biofouling and the degradation performance of the sheets.

## Introduction

Nowadays, our world is facing a great deficiency in water resources due to the increasing population, fast growth, and the abuse of natural resources^1^. United Nations (UN) recognized ensuring water security as one of the sustainable development goals^2^. One of the most serious environmental problems, is the presence of synthetic dyes in industrial wastewater effluents, resulting from many industrial processes like tanning and textile industries. The existence of dyes in wastewater may lead to serious problems for human beings as well as aquatic life, even if found in low concentrations^3^. Recent studies indicated that pollutants in wastewater released as a result of industrial activities harm aquatic organisms in water environments^4^. Methylene blue (MB) is considered one of the cationic dyes that are utilized in coloring paper, and dyeing cotton, wool, silk, and leather. However, its extensive use can cause eye burns, breathing difficulty, nausea, methemoglobinemia, increasing sweating, and mental confusion^5,6^. Moreover, crystal violet (CV) is also a cationic, tri-phenylmethane, dye that was commonly used to stain wool, silk, and cotton products into purple with a color ranging from blue to red. In addition to being used in human & veterinary medicine as a biological stain. However, its excessive use can cause eye, skin, and digestive tract irritation, vomiting, heart disease, quadriplegia, and induce carcinogenesis in humans^7,8^. Furthermore, Congo red (CR) is an anionic diazo dye that has widespread applications especially in the textile industry. CR is a highly toxic and carcinogenic dye that has strong resistance to discoloration & biodegradation^9,10^. CR has been banned by several countries, but it is also still widely used in others.

That is why, it is essential to remove these dyes from industrial wastewater effluents prior to pouring them into the water stream. Several chemical, physical, and biological techniques are usually used for removing dyes from wastewater. These techniques include adsorption, coagulation, membrane separation, softening electrochemical, flotation, as well as reverse osmosis^11–16^. Nevertheless, most of these methods that are used, have some drawbacks like their excessive energy demand, expensive operation cost, slowly removing process, besides the production of hazardous byproducts^17^. Therefore, searching for a cheap and environmentally safe technique such as biosorption became a point of concern to many researchers, which is considered a process of using natural biomasses like agricultural wastes, fungi, and algae, as bio-sorbents for dyes^18–20^. Marine algae (seaweed) is considered as one of the likely biosorbent materials due to the incorporation of alginate gel in the cell wall in addition to polysaccharides that contain (different sites for binding, for example: hydroxyl, carboxyl, sulfonate as well as amine groups)^21,22^.

*Sargassum* biomass has been used in the removal of heavy metals and organic dye from wastewater effluents^23–25^. The limitation in the applicability of the capacity of adsorption of the biomass of algae, in its natural form, was due to its low surface area which leads to a low van der Waals interaction. Grinding of the biomass of algae, to increase the surface area & accordingly its active functional group is a favorable approach for increasing the adsorption capacity. In addition to it can be incorporated and distributed within a porous matrix for obtaining applicable form during use^26^. Polymers are a kind of promising materials that might be easily formed in various shapes such as fibers, beads, and flat sheets having a controlled porosity and morphology. Polyethersulfone (PES) polymers have been widely used as attractive materials because of their great stability: thermal, oxidative, and chemical wise, also due to their outstanding mechanical properties^27,28^. The hydrophobic property of PES limits its widespread application because of its biofouling problem^29^. Therefore, the main aim of our current work is to present a facile approach for wastewater treatment using cost-effective and eco-friendly new biosorbent-materials based on *Sargassum dentifolium* (S) biomass incorporated into porous polyethersulfone (PES) matrix for ease processing during application. The composite matrix was prepared using simple process that can control porosity and morphology. Zinc oxide (ZnO) nanoparticles as an anti-biofouling agent were added to the polymer sheet forming a composite sheet (PES-S-ZnO). Different characterization methods were applied for the composite sheets like ATR-FTIR, SEM, EDX, Swelling & Porosity measurements. The PES-S-ZnO sheets were used to remove the MB, CV and CR dyes from synthetic wastewater. The removal processes were optimized using One-Factor- at-a-Time (OFAT). Langmuir, Freundlich, Temkin, and Dubinin–Radushkevich isotherm`s models were assessed for the detection of the dye’s adsorption mechanism. Moreover, the removal process kinetics, Pseudo first-order, second order, and intraparticle diffusion models, were evaluated, to obtain the dye adsorption rate using the composite sheets. The composite sheets were tested for natural degradation using an accelerated weathering test. Moreover, the anti-biofouling test was examined for the composite sheets using microbial strain enriched from real industrial water samples.

## Materials and methods

### Materials

Polyethersulfone was acquired from BASF, Germany. N, N Dimethyl formamide (DMF) as a solvent which was supplied by British Drug House (BDH) company, USA. Crystal violet (CV), and Methylene blue (MB) dyes were delivered from RANKEM chemicals. Congo red was acquired from S.D. fine chemicals Ltd, India. Acetone, ethanol, hydrochloric acid, were acquired from Fisher chemicals. Ltd. Polyvinyl pyrrolidone (PVP) (M.wt; 40 KDa) was supplied from Merck. Zinc oxide (ZnO) nanoparticles with a particles size ranged from 7 to 40 nm were previously prepared by Dahran et al. using Moringa leaves aqueous extract^30^.

### Methods

#### Algal biomass collection and grinding

Marine macro-algal specie *Sargassum dentifolium* (S) was collected from Ras Gharib, Red Sea, Egypt, and washed by de-ionized water then oven dried at 50 °C in for 2 h. After that, the dried algal biomass was ground using Ball Mill (Planetary Ball Mill, PM 400 “4 grinding stations) to a micro-size (~ 10 µ).

#### Composite sheets preparation

Using a phase inversion technique, composite algal polyethersulfone (PES) sheets were prepared^31^. The PES powder was added to N, N Dimethyl formamide at a concentration of 15% w/v till complete dissolution to form 15% w/v polymer solution. Polyvinyl pyrrolidone 10% (PVP) was applied to the solution to enhance the porosity of the sheets on casting. Polymer solutions were mixed with various ratios of the ground alga ranging from (0, 10, 20, 30, 40 and 50%). Then, Zinc oxide (ZnO) nanoparticles were added to the solution, as an anti-biofouling agent, at different concentrations (0.5, 1.0, 2.0 and 4.0%). After mixing all the solutions of the dried macro-algae, they were dispersed via an ultrasonic water bath for 1 h, in order to disperse the dried biomass. The resulting homogenous solution was then poured on a glass plate, that was cleaned and dried, and cast using a film applicator adjusted to produce 250 μm thick sheet. The glass plate together with the casted solution was directly immersed in a coagulation water bath for 1 h to initiate polymerization. The sheets were then separated from the plates, washed with distilled water and finally dried.

#### Characterization of the composite sheets

##### Attenuated total reflectance Fourier transform infrared (ATR-FTIR)

Attenuated Total Reflectance Fourier transform infrared (ATR-FTIR) spectroscopy was used to, estimate the changes detected in the vibration frequency in the functional groups of the biosorbent composites. The sheets have been dried overnight before measuring the vibration frequency changes within the spectral range 400 to 4000 cm^− 1^.

##### Scanning electron microscopy (SEM)

The surface morphology of the composite sheets and their elemental analyses were explored using high-resolution scanning electron microscope (QUANTA FEG 250, ESEM, using accelerating voltage of 200 V–30 kV) and connected to Energy Dispersive X-ray Microanalysis (EDX). Prior the examinations, the prepared PES, and PES-S films were eventually sputter-coated with gold vapor, via using S150A Sputter Coater-Edwards.

##### Swelling performance

Swelling behavior of the composite sheets of 2 × 2 cm^2^ was assessed by immersing them in distilled water for a period of 24 h, at room temperature. Wet sheets’ weights, were recorded, followed by drying them at 60 °C under vacuum until constant weights are obtained^32^. Equation no. (1) was used to estimate the percentage of swelling:

1 $${\text{Swelling}}\;\% ={\text{ }}[{{\text{W}}_{\text{2}}} - {{\text{W}}_{\text{1}}}/{{\text{W}}_{\text{1}}}]*{\text{1}}00$$

W_1_ and W_2_ represent the dried weight and wetted weight of sheets, respectively.

##### Porosity properties

The porosity of the composite sheets was estimated based on the dry–wet weight procedure^33^. The wet weights of the sheets were reported after removing the excess water by sandwiching them briefly between a pair of dry filter papers. The swelled sheets were dried in an oven at 60 °C for a day then the weight was reported. Afterwards, the porosity of the sheets was estimated according to the following equation:

2 $${\text{e}}\% =({\text{Wwet}} - {\text{Wdry}})/{\text{dwAh * 1}}00$$

Where ε: the porosity of the sheet, dw: the water density (0.998 g/cm^3^), A: the area of the wet sheets (cm^2^ and h: the thickness of wet sheets (cm).

##### Assessment of point of zero charge of the composite sheet

The point of zero charge (pH_pzc_) for the composite was assessed via using pH drift manner according to stated literature^12^. Briefly, the pH of KCl solution (5%) was adjusted to a value ranged from 2 to 10 using HCl (0.1 M) or NaOH (0.1 M). The adsorbent composite (0.05 g) was added to 20 mL of each adjusted pH solution in a capped bottle under mild shaking for about 24 h. The final pH was reported and the difference between the final and initial pH was determined and plotted against the initial pH. The pH_pzc_ was determined from the curve at ∆pH equal zero.

##### Leaching of ZnO nanoparticles from polymer algal sheet

The percentage of leaching was measured by exposing ZnO impregnated polymer sheets to aqueous solutions of various pH values. The percent of the ZnO nanoparticles, leached into the solution was evaluated using atomic absorption spectroscopy, flame unit of atomic absorption spectrophotometer (AA, Model- M series Thermo scientific, National Institute of Occupational Safety and Health (NIOSH).

#### Optimization of dye removal

The removal of dyes from aqueous solutions using composite sheets was optimized by changing One-Factor-at-a-Time (OFAT). The tested factors included pH values (3–11), contact time (0–24 h) and initial dye concentrations (100–500 mg/l), using various types of PES sheets with different ratios of *Sargassum* using increasing dose of the sheet 1.25, 2.5, 5 and 7.5 g/l. Dyes concentrations were determined using UV-Visible spectrophotometer HACH (DR 3900) at 660, 590 and 502 nm for Methylene blue, Crystal violet and Congo red, respectively. The removal of dye efficiency was demonstrated based on the next equation:

3 $${\text{Removal}}\;{\text{efficiency}}\;\% ={\text{ }}[{{\text{C}}_{\text{o}}} - {{\text{C}}_{\text{e}}}/{{\text{C}}_{\text{o}}}]~{\text{1}}00$$

Where C_o_ and C_e_ are considered the initial and final dye’s concentrations, respectively.

Whereas the adsorption capacity (qe: mg/g) of the composite sheets that can be calculated using the following equation:

4 $$\:qe=\frac{Co-Ce}{Ce}X\:V$$

Where V (L) is considered the volume of dye solution.

#### Adsorption isotherm

Linear Langmuir and Freundlich isotherms were used to determine the mechanisms by which biosorption of CV, MB and CR take place.

##### Langmuir isotherm

Langmuir isotherm was used to determine the mechanism of dyes biosorption by the biosorbent material^34^. The Langmuir isotherm is expressed by the following equation:

5 $${{\text{C}}_{\text{e}}}/{{\text{Q}}_{\text{e}}}={{\text{C}}_{\text{e}}}/{{\text{Q}}_{{\text{max}}}}+{\text{1}}/{\text{b}}{{\text{Q}}_{{\text{max}}}}$$

Q_e_, represents the quantity of sorbed dyes per unit weight of sheets (mg/g), Q_max_, represents the maximum adsorption capacity (mg/g), and b is Langmuir constant which related to the adsorbate the adsorbent affinity (l/mg).

##### Freundlich isotherm

Freundlich isotherm is an empirical equation, which is needed in the estimation of the biosorption intensity of the sorbent towards the biosorbent sheets via the following equation^35^.

6 $${\text{Log}}\;{{\text{q}}_{\text{e}}}={\text{Log}}\;{{\text{K}}_{\text{f}}}+1/{\text{n}}\;{\text{Log}}\,{{\text{C}}_{\text{e}}}$$

Where qe, is considered the biosorption capacity at equilibrium (mg/g), Ce is considered the concentration of dye in the solution (mg/l), while Kf, is empirical Constant represent the biosorption capacity (mg/g), and n, is known to be the degree of dependence of adsorption with equilibrium concentration.

##### Temkin isotherm

Temkin isotherm is a model based on the pre-assumption that the heat of adsorption would diminish directly with the raise in covering of adsorbent^36^. The straight type of this isotherm was represented by the following equations:

7 $${q_e}=\left( {{{RT} \mathord{\left/ {\vphantom {{RT} {{b_T}}}} \right. \kern-0pt} {{b_T}}}} \right)\ln {a_T}+\left( {{{RT} \mathord{\left/ {\vphantom {{RT} {{b_T}}}} \right. \kern-0pt} {{b_T}}}} \right)\ln {C_e}$$

8 $${q_e}=B\ln {a_t}+B\ln {C_e}$$

9 $$B=\frac{{RT}}{{{b_T}}}$$

Where, b_T_ (mg /L) is the Temkin constant, a_T_ (l/g) is a constant at equilibrium.

##### Dubinin–Radushkevich isotherm

The Dubinin–Radushkevich (D–R) isotherm is frequently used to evaluate the adsorption mechanism and assess the porosity performance of the adsorbent. The D–R isotherm model assumes that the adsorption is happen in the porous material and assesses the adsorption apparent energy^37^. The direct equation of D–R isotherm is given by the following equation:

10 $$\ln {q_e}=\ln {q_{\hbox{max} }} - \left( {{K_{ads}}{\varepsilon ^2}} \right)$$

Where, q_e_ is the adsorption capacity at equilibrium (mg /g), K_ads_ is the D–R constant that recognized as the mean free energy of adsorption per mole of adsorbate (J/mol) and $\:\epsilon\:$ is the polanyi potential determined by the next Equation:

11 $$\varepsilon =RT\ln \left( {1+{\raise0.7ex\hbox{$1$} \!\mathord{\left/ {\vphantom {1 {{C_e}}}}\right.\kern-0pt}\!\lower0.7ex\hbox{${{C_e}}$}}} \right)$$

Where, R is universal gas constant (8.314 J /mol K) and T is the temperature in Kelvin. By plotting lnq_e_ versus $\:\epsilon^{2}\:$ a straight line formed with a slope equal K_ads_ and intercept equal ln q_max_.

The D-R model is mainly used to estimate the average free energy of adsorption (kJ mol^− 1^):

12 $$\:E=1/\sqrt{2Kads}$$

#### Kinetic studies

Kinetics studies were conducted, in order to estimate the rate of dye biosorption on the composite adsorbent material. Kinetics models are famous to follow either pseudo first-order or pseudo second-order equation^38^.

The pseudo first order model follows the following equation:

13 $${\text{log}}({{\text{q}}_{\text{e}}} - {{\text{q}}_{\text{t}}})={\text{log}}{{\text{q}}_{\text{e}}} - {{\text{K}}_{\text{1}}}{\text{t}}/{\text{2}}.{\text{3}}0{\text{3}}$$

Where k_1_ (1/min) is the rate constant of pseudo first order, and q_t_ (mg/g), the amount of sorbed molecules per unit mass at time t (min), respectively.

Pseudo second order models follow the following equation. 

14 $${\text{t}}/{\text{qt}}={\text{t}}/{{\text{K}}_{\text{2}}}{{\text{q}}_{\text{e}}}^{{\text{2}}}+{\text{t}}/{{\text{q}}_{\text{e}}}$$

Where k_2_ (g/mg min) is the rate constant for pseudo-second order and q_e_ (mg/g) is the quantity of sorbed material at equilibrium.

The intraparticle diffusion model (IPD) proposed by Weber and Morris has been widely applied for the analysis of adsorption kinetics. Weber and Morris discovered that in many circumstances of adsorption, solute uptake is almost proportional to t^1/2^ rather than to the contact duration t^39^ see Eq. (15).

15 $${\text{qt}}={{\text{K}}_{{\text{int}}}}{{\text{t}}^{{\text{1}}/{\text{2}}}}$$

Where K_int_ is intraparticle diffusion rate constant. A plot of qt versus t^1/2^ should be a straight line with a slope of K_int_ when intraparticle diffusion is the rate-limiting step. The straight line should pass through the origin if intraparticle diffusion is the sole mechanism for adsorption.

#### Thermodynamics of adsorption

To evaluate the thermodynamic parameters, i.e., the Gibbs free energy (ΔG^o^), enthalpy change (ΔH^o^) and entropy change (ΔS^o^); adsorption was achieved at different temperature to assess the feasibility and nature of the adsorption process^4^. The change in Gibbs free energy of the adsorption is correlated to the equilibrium constant (K_d_) by the following equation. 

16 $${\text{D}}{{\text{G}}^{\text{o}}}= - {\text{RT}}\;ln\;{{\text{K}}_{\text{d}}}$$

17 $${\text{ln}}{{\text{K}}_{\text{d}}}={\text{D}}{{\text{S}}^{\text{o}}}/{\text{R}} - {\text{D}}{{\text{H}}^{\text{o}}}/{\text{RT}}$$

Where R is the gas constant (8.314 J/mol.K) and T is the temperature in Kelvin, K_d_ is distribution coefficient that can be assessed from the next Eq. 

18 $${\text{Kd}}={\text{Qe}}/{\text{Ce}}$$

Where Qe is the adsorption capacity at equilibrium (mg/g) and Ce is the concentration at equilibrium (mg/L). The values of ΔH◦ and ΔS◦ were assessed from the slope and interception of the plot of ln K_d_ verse 1/T, respectively. The value of ΔG◦ can be calculated using the following equation:

19 $$\Delta {{\text{G}}^{\text{o}}}=\Delta {{\text{H}}^{\text{o}}}--{\text{T}}\;\Delta {{\text{S}}^{\text{o}}}$$

#### Regeneration studies

Recovery studies of the composite sheets were carried out using (1:1) ethyl alcohol: distilled water. After being adsorbed, methylene blue, crystal violet and Congo red on the composite sheets, the sheets were soaked in fresh recovery solutions for 3 times till no apparent color was observed followed by the washing using distilled water and drying. The adsorption and recovery processes were repeated for 5 cycles followed by calculating the removal percentage.

#### Anti-biofouling evaluation of the composite sheets

Untreated water sample, from a dying and preparation factory at the 10th of Ramadan city, Egypt. The apparent color of the collected water sample indicated that it contained high concentrations of dyes. The values of both biological oxygen demand (BOD) and chemical oxygen demand (COD), of the collected sample were 182 mg/L and 668 mg/L respectively. The sample was enriched with Mueller Hinton broth (MHB) media (Difco, Franklin Lakes, NJ, USA) and then incubated at 35 °C overnight in a shaking water bath at 200 rpm. Serial dilution method was used to isolate the most predominant bacterial from the water sample to be used as a test organism for assessing the anti-biofouling properties of ZnO.

The purified organism was identified using 16 S rRNA^40^. The PCR product was sequenced by Eurofins (MWG GmbH, Germany). The sequence was then analyzed using BLAST and FASTA programs in order to construct a phylogenetic relationship using Tree View software. The resulting pure colonies were sub-cultured on 10 ml MHB to an optical density (OD) of 0.2 at 550 nm and incubated in a shaking water bath (200 rpm), at 35 °C for 3–4 h until an OD550 of 1–2 was obtained. After that, the MHB cultures were then diluted 100 folds to achieve bacterial counts of 1-2 × 10^8^ CFU/ml (Colony-Forming Unit/ml) according to the Clinical Laboratory Standards Institute (CLSI)^41^. Aliquots of 0.2 ml of 100-fold diluted bacterial suspensions from the above-mentioned culture were used for filling 14 wells out of 2 plates (Nunc GmbH & Co., Wiesbaden, Germany) while filling the remaining 2 wells with sterile MHB as a negative control. The sheets samples of a dimension (1.0 × 1.0 cm^2^ were then cut and placed at the bottom of the 12-well plates. The sheet`s samples were arranged as: Two wells for blank sheets (PES without biomass and ZnO), two wells for PES-S sheet without ZnO, two wells for each PES-S-ZnO with different ZnO concentrations (0.5, 1.0, 2.0, and 4.0%). The plates cultivated at 35 °C overnight with a continuous agitation at 200 rpm. Afterwards, the plates OD were tested at 550 nm in comparison to the negative control. The result was introduced as an average ± standard deviation (SD).

#### Accelerated weathering test of the polymer sheets

The composite sheets were subjected to weathering using an accelerated weathering tester model QUV/se (Q-LAB, Westlake, OH, USA). The ASTM D6164 Cycle-C weathering conditions used. Under 100% condensing humidity, fluorescent UV lamps (UV-A-340) with 0.76 W m^2^ irradiance (wavelength 340 nm) were used in cycles of 8 h of UV irradiation at 50 °C followed by 4 h of darkness at 50 °C. These uninterrupted cycles were carried out on specimens affixed to test panels. The effects of accelerated weathering were evaluated for 100 h of exposure periods. After weathering treatment of each sheet, the sheets were collected and compared with untreated ones. Visual changes were monitored and were found to appear as brownish color. Increasing the color intensity was used an indicator for weathering^42^.

## Results and discussion

### Composite sheet characterization

#### Fourier transform infrared (ATR-FTIR)

**Fig. 1:**
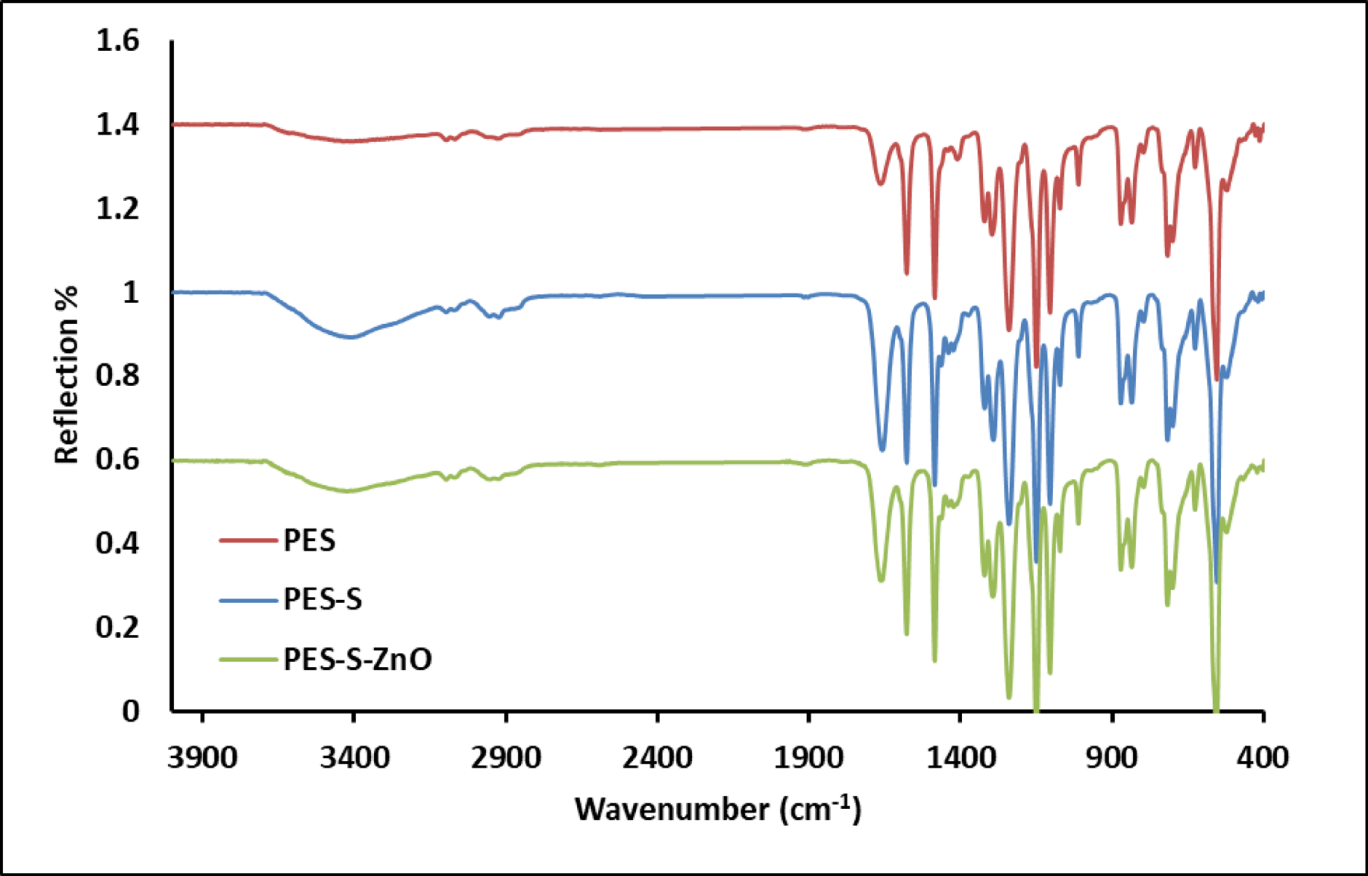
ATR-FTIR spectrum of the PES, PES-S and PES-S-ZnO sheets.

PES, PES-S and PES-S-ZnO sheets were evaluated using ATR-FTIR as demonstrated in Fig. 1. By comparing the ATR-FTIR spectra of the prepared sheets with and without the addition of algal biomass and algal biomass with ZnO it could be concluded that the peak of 3445 cm^− 1^ which is related to O-H stretching of polysaccharide of algal cell wall which may enhance the hydrophilicity of the composite sheets. The aliphatic peaks at 2923 and 2850 cm^− 1^ were clearly appeared which represent the CH and CH_2_ of aliphatic of polysaccharide molecules of algal biomass, phenolic compounds and phospholipid^43^. The peak at 1660 cm^− 1^ was attributed to the carboxylate groups of algal biomass. Addition of ZnO nanoparticles did not change largely the FTIR spectra of the polymer composite, however there are a decrease in the intensity of hydroxyl groups appeared around 3400 cm^− 1^ which represent formation of hydrogen bonding between them, was obvious in the sheets containing algae (PES and PES-ZnO). The bonding of algal biomass and PES polymer are mainly physical bonds, like hydrogen bonding or/and Van Der Waals interactions which help in understanding the reason for the varying affinities of the impregnated sheet against the tested cationic and anionic dyes. On carrying out ATR-FTIR it was observed that PES-S spectra, broad peak around 3445 cm^− 1^ attributing the capability of *Sargassum* impregnated sheet to recover the studied dyes^44^.

#### SEM analysis

**Fig. 2:**
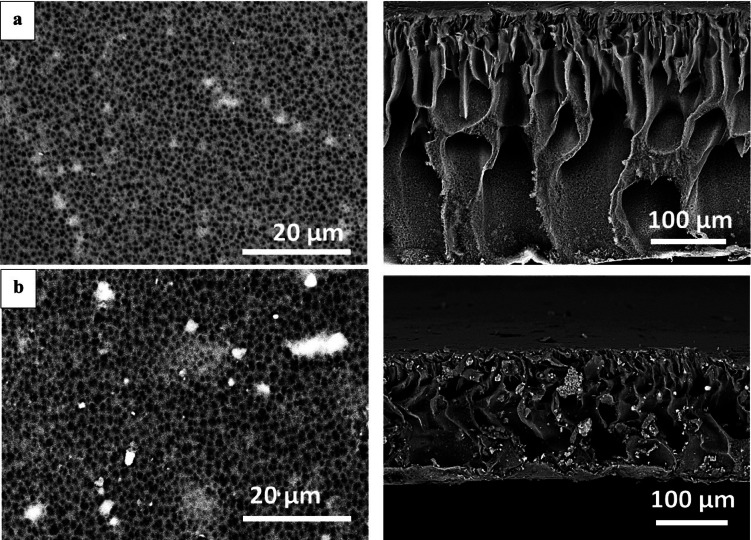
SEM of sheets surface (left) and cross section (right) for (**a**) PES, and (**b**) PES-S, respectively.

Surface morphologies of the PES and PES-S and sheets were investigated using scanning electron microscopy (SEM) analysis as demonstrated in Fig. 2 (left). The SEM result illustrated that the PES sheet had a smooth surface with rather small pores. whereas the PES-S-20% composite sheet demonstrated relatively rougher surface with larger numbers of pores. Figure 2 (right), represented the SEM cross section of the sheets. The PES blank sheet pointed out a smooth edge with finger like structure with macro-voids beneath and thin dense top layer structure^14^. By embedding the micro-particles of *Sargassum dentifolium* algal biomasses; the cross section showed highly rough construction with increased the macro-void size and presence of micro-particles of algal biomass. The incorporation of the micro algal biomass led to decrease in the polymer concentration which resulted in lower viscosity of the casting solution that form film with thinner thickness and large macro-void structure^45^.

**Fig. 3:**
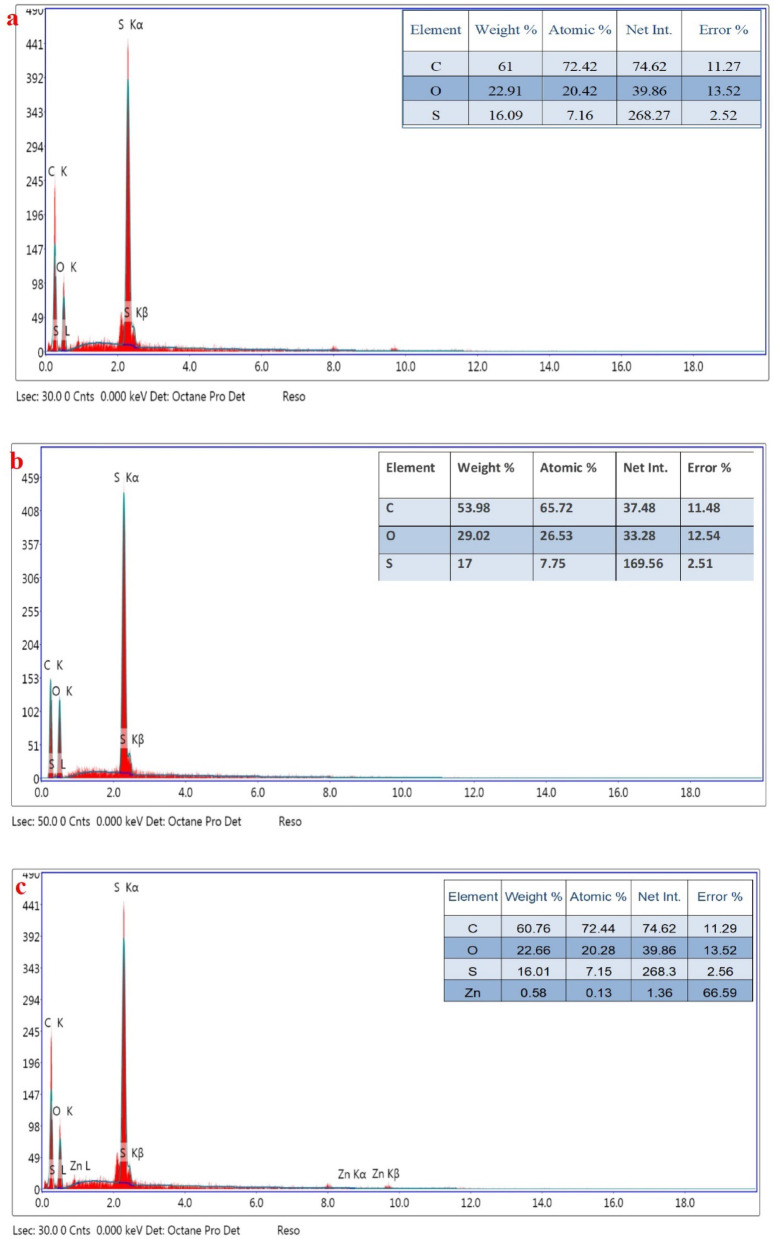
EDX of PES (**a**), PES-S (**b**) and PES-S-ZnO (**c**) sheets, respectively.

**Fig. 4:**
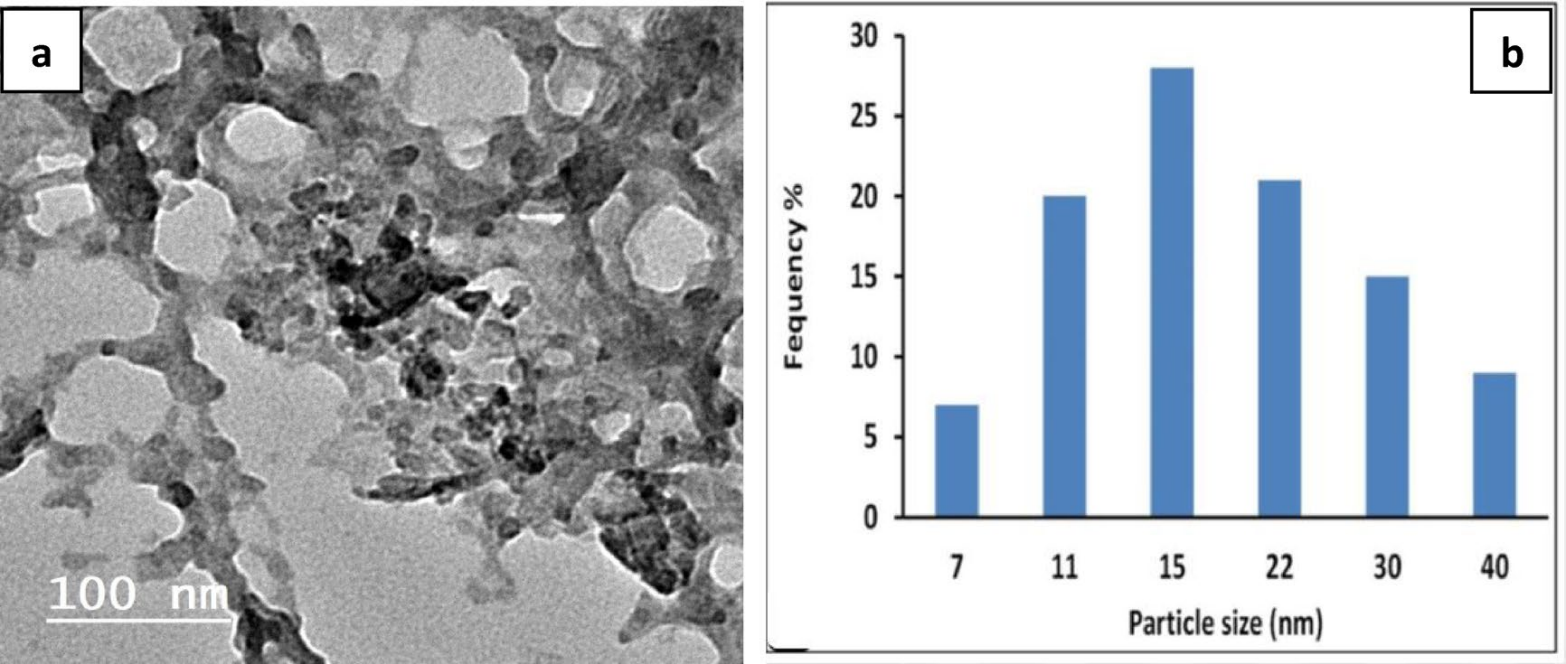
TEM of synthesized ZnO nanoparticles (**a**) and particles size frequency obtained from TEM image using ImageJ program (**b**).

The elemental analysis of the composite sheets was explored using Energy dispersive X-ray (EDX) as displayed in Fig. 3. The results displayed the presence of Zn element that indicate the fixation of ZnO nanoparticles in the polymer matrix. The presence of sulfur element was a result of the structure of polyethersulfone and sulfate groups in the incorporated algal biomass^44^. ZnO nanoparticles was previously synthesised using Moringa extract and full characterization was attained and reported in the published paper Dahran, et al., (2023)^30^. The particle size and shape of the obtained ZnO were assessed by using measurements transmission electron microscope (TEM) (HR-TEM, JEM-2100, JEOL, Tokyo, Japan), and the results were displayed in Fig. 4a. One can see that the obtained particles were in semi-spherical shape within the range of 7–40 nm. The particle size of ZnO was evaluated using ImageJ software analysis, as indicated in Fig. 4b. The main prominent size of the particle was about 15 nm. Some particles aggregate into clusters comprising of tens or hundreds of separated nanoparticles, forming rod-like structures.

#### Swelling and porosity

**Fig. 5:**
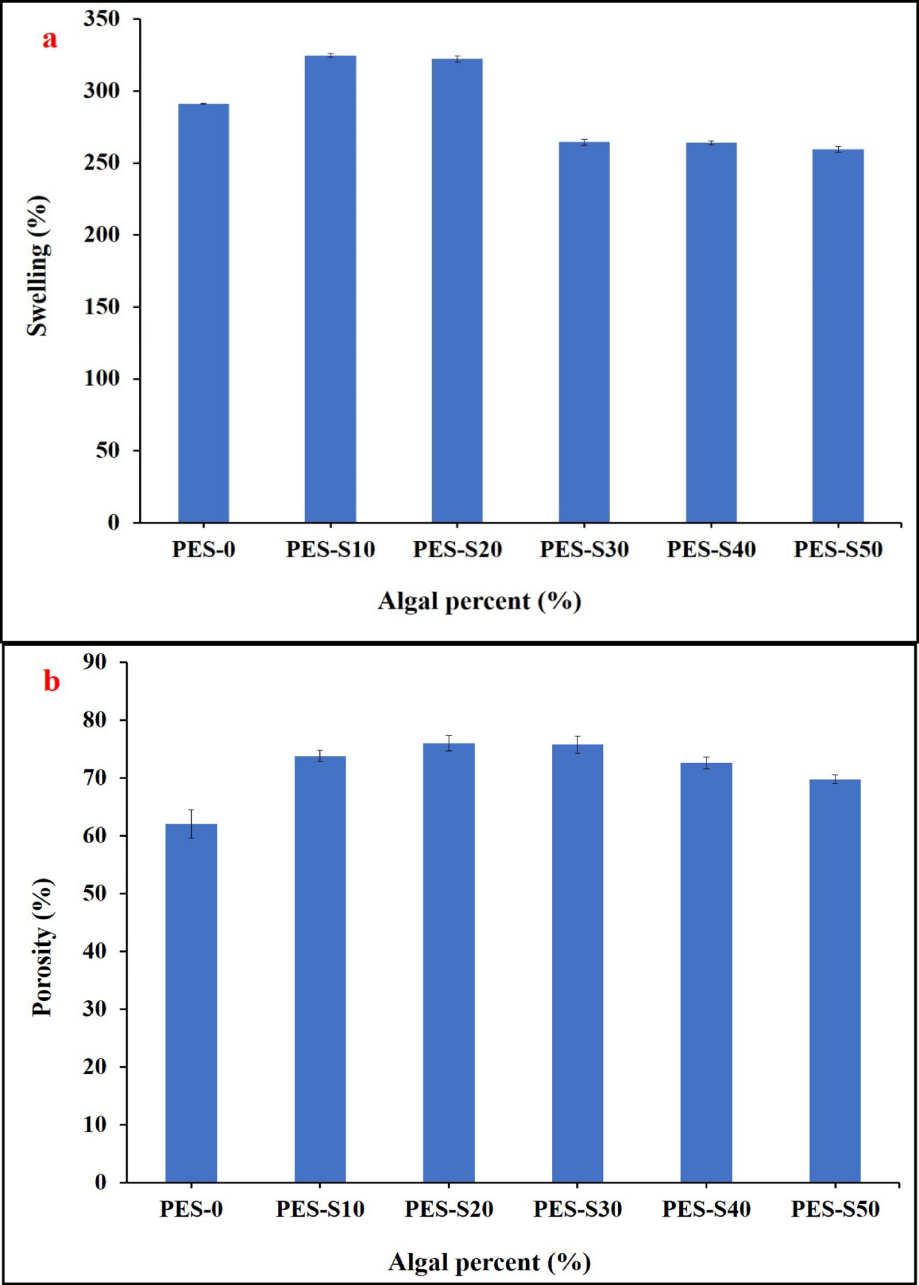
Swelling (**a**) and porosity (**b**) behavior of the PES sheets with different ratios of *Sargassum dentifolium* (S) of 0, 10, 20, 30, 40 and 50%.

The swelling of the polymer sheets with and without the algal biomass was explored and displayed in Fig. 5a; it can be observed that the swelling behavior of the sheet increased from 291.5 to 324.8% on adding *Sargassum dentifolium* biomass at a ratio of 10%. At the algal percent of 20%, the swelling was decreased slowly to 322.3%. Further increase in the algal biomass didn’t affect the swelling performance of the sheets. The enhanced increase of the swelling of the composite sheet may be due to the presence of hydrophilic reactive functional groups of the biomass cell wall structure that contains polysaccharide phenolic compounds and proteins e.g., OH, C = O, COOH, SO_3_- and others^36,44^.

The porosity of the sheets was also assessed gravimetrically and the results were illustrated in Fig. 5b. The porosity of the PES blank sheet was around 62.0% and increased to 73.68% by adding *Sargassum dentifolium* biomass at a ratio of 10%. The porosity was further partially increased to 76.04% of 20% algal biomass. Further increase in the algal biomass to 30% the porosity was slowly decreased to 75.8%. Afterwards, the porosity was decreased to 72.65% and 69.8% of 40 and 50% algal biomass, respectively. These results may be related to the hydrophilic character of algal micro-particles and decreased viscosity that may enhance the macro-void formation of the sheet during preparation process^46^. The improvement of the swelling and porosity characteristics predicted a good removal efficiency of dyes using the composite sheets. These results in agreement with Peng et al.., 2021 who investigated that the initial increase of swelling and porosity may be related to the decreased in viscosity of the film forming polymer (PES) due to increase the algal micro-particles which led to increase in the macro‐void formation of the sheet during preparation process^47^. Whereas further addition of algal micro-particles to the polymer casting solution relatively decreases swelling and porosity may be the aggregation and accumulation of these high concentrated microparticles and the low chance of well distribution through the polymer matrix^48^.

#### Point of zero charge of the composite

**Fig. 6:**
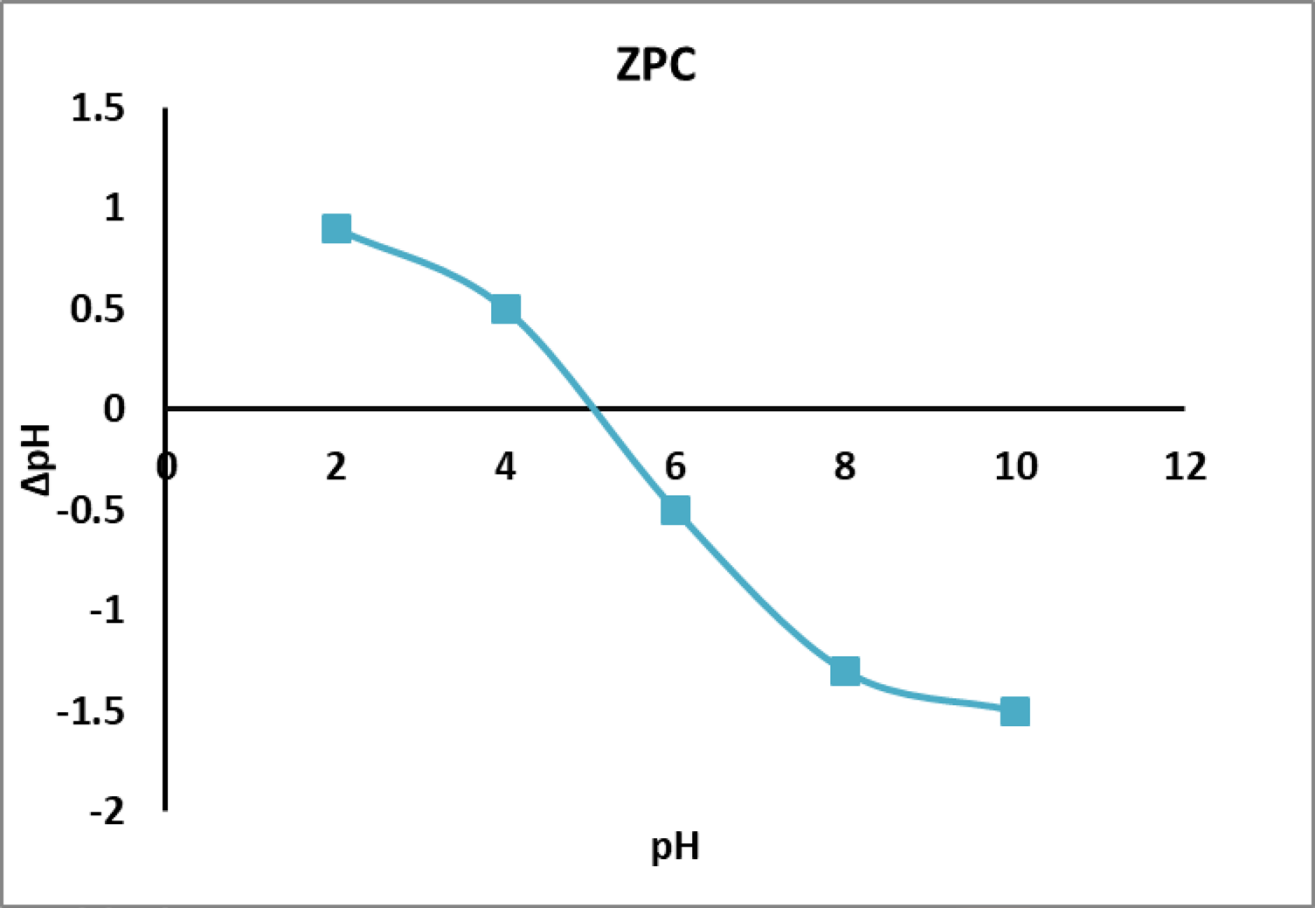
Zero point of charge (pH_pzc_) for PES-S-ZnO composite sheet.

The result of the point zero of charge was displayed in Fig. 6 and showed that at pH 5.5; the composite have no changes with indicate that after this point the composite have negative charge which favor removal of cationic molecules and below this point (less than 5.5) the composite carry positive charge. However the result of pH_pzc_ was obtained after two days, during first two days there no change of pH before and after this experiment for all tested pH. However the adsorption governed not only by surface charge but also the hydrophobic interaction between the aromatic rings of dye molecules via π–π stacking phenomenon and intra-diffusion of the dye molecule into the interior pores of the composite which can be improved by increasing the pH of solution which breakdown the intra-hydrogen bonding of the composite which resulted in enhancing the adsorption of anionic dye into the composite^49^. Also there are hydrogen bonding and weak vander waals attraction forces.

#### Leaching of ZnO nanoparticles from polymer algal sheet

The result was summarized in Table 1. The maximum leaching of ZnO nanoparticles was obtained at an acidic solution (pH ~ 3) whereas about 10% ZnO was leached after 6 days contact time. The leaching at neutral and basic conditions was minor, reaching about 5.48 and 4.58% after 6 days of contact for the pH values of 5.5 and 9.5, respectively. These results indicated that low percent of ZnO was leached from the composite sheets which gave an indication of a relative stability of these nanoparticles within the polymer composite^50^.

**Table 1:** Leaching of ZnO nanoparticles from the composite sheets at different pH values.

pH values	1st day (%)	2nd days (%)	6th days (%)
3	7.50	9.32	10.75
5.5	1.80	3.75	5.48
9.5	1.05	1.35	4.58

### Adsorption studies

#### Effect of algal dose on the adsorption process

**Fig. 7:**
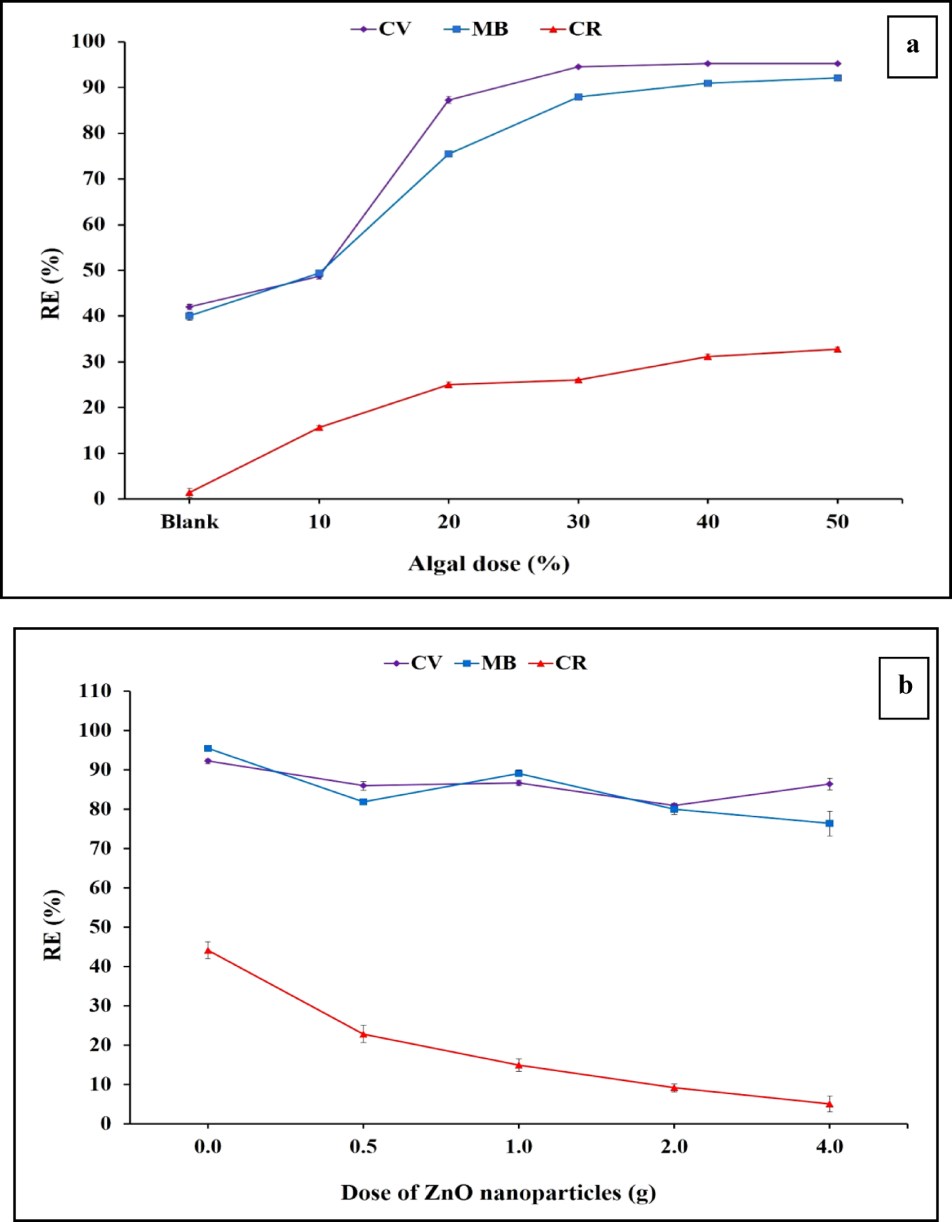
Effect of different algal doses (**a**) and ZnO concentration (**b**), within the sheets on removal efficiency of the tested dyes.

The effect of the incorporation of algal biomass into polyethersulfone sheet on the adsorption of different dyes was tested and results are shown in Fig. 7a. The efficiency of dye removal upon the increased dose of algal biomass starting from 10 to 50% was assessed. The results indicated that by increasing the algal dose, the adsorption of crystal violet increased from 42% for PES to 95.26% for PES-S, while the incremental of methylene blue increased from 40 to 91.52%, moreover; the adsorption of Congo red increased from 1.39 to 51.45%. Due to mechanical strength deterioration on increasing the algal biomass, 20% dose was selected as an optimum concentration for dye removal. The increase in the CV removal efficiencies may resulted from the active functional groups of the algal biomass. The effect of different algal doses on the adsorption of MB dye was also studied. For the all-dyes adsorption, it was noted that over 20% of an algal dose didn’t significantly enhance the removal efficiencies. The adsorption of CR was greatly lower than MB and CV. This result may be due to the adsorption mechanism of the cationic dyes (CV and MB) by the negatively charged algal biomass functional groups e.g., carboxylate, hydroxyl and sulfonate groups^51^. Further optimization of adsorption process was continued using (20%) ratio of *Sargassum* within the polymer sheets.

#### Effect of ZnO nanoparticles on the adsorption process

Figure 7b. shows the effect of adding various doses of antifouling ZnO nanoparticles on the adsorption process of the dyes under investigation. There was a negligible effect on the investigated dyes’ adsorption on impregnating the polymer sheet with 0.5 or 1% ZnO nanoparticles. However, higher doses of ZnO decreased dyes removal at a rate ranging from 2 to 12% ^52^. Therefore, the concentration of 1.0% ZnO nanoparticles was selected for the further experiments. These results are in agreement with (Alanazi, 2023) who investigated the efficiency of removing malachite green utilizing composite of graphene oxide-zinc from aqueous solution^53^.

#### Effect of contact time on the adsorption process

**Fig. 8:**
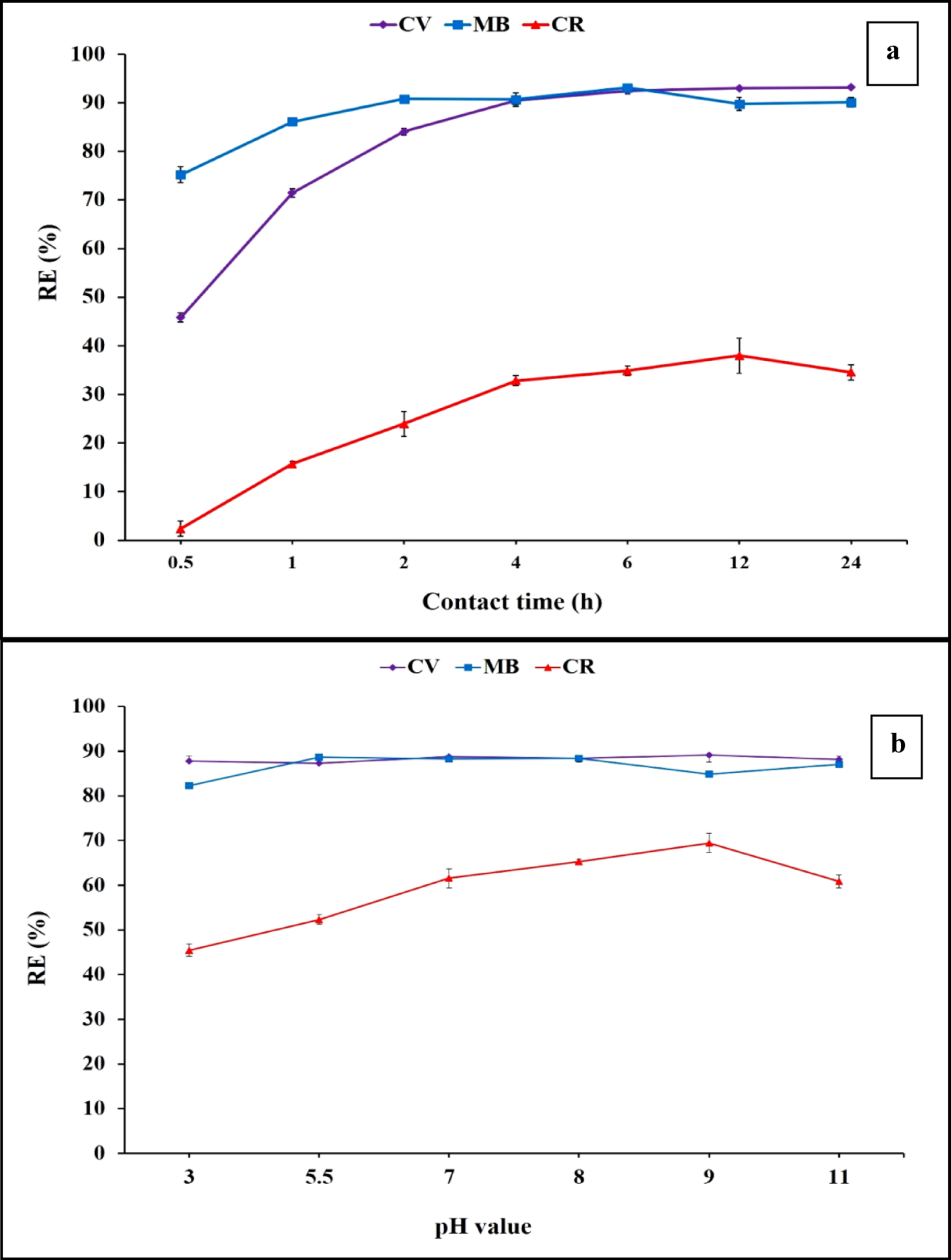
Effect of different contact time intervals (**a**) pH values (**b**) on removal efficiency of the tested dyes.

Different contact times (0.5–24 h) were explored for CV, MB and CR removal. Composite sheets made of polyethersulfone polymer impregnated with the adsorbent and the antifouling agent was used at optimized doses of 20% and 1.0%, respectively. The first evaluation was in a static mode with 200 ppm dye concentration and pH value of 5.5. Figure 8a illustrates the dye removal by the composite sheets, at which maximum removal efficiencies of 92.46, 86.1 and 34.54% were achieved after 6, 6 and 12 h for CV, MB and CR, respectively. These results can be ascribed to the negative nature of the composite sheets which correlated to its negative functional groups that are found in the phenolic compounds, polysaccharides, carotenoids, proteins, and lipids^36,46^. These functional groups are represented in carboxylate, hydroxylate, amine, sulfonyl, and carbonyl groups that have anionic character and have high affinity for the cationic dye-binding process as previously reported^21^. The mechanism of the algal biomass`s dye adsorption was previously described and correlated to many factors as follows; (i) the interaction between the cationic dye and the negatively charged functional groups, (ii) the coordinate bonds that may be arised from the electrons lone pair of electron rich groups such as OH group or NH_2_ and the dye molecules, (iii) Van der Waals forces that may be formed between nonpolar groups of algal cell wall molecules and dye molecules and (iv) ion–dipole bond that may be achieved between dye`s molecules and the negatively charged dipole end of the carbonyl group. By increasing in contact time, the adsorption capacities also increase until reached a constant value. These changes in the rate of adsorption may be due to that at beginning the initial adsorbent sites are vacant and available for adsorption process and with continued time a saturation level was attained as previously reported^13^.

#### Effect of pH values on the adsorption process

Different pH values were investigated for the PES-S-ZnO against CV, MB and CR removal and the results were represented in Fig. 8b. It was observed that there is no significant difference regarding removal efficiencies of CV & MB at different pH values. On the other hand, the influence of changing pH values for the Congo red removal followed by a pattern of dye precipitation at pH (3), followed by increase in removal efficiency up to pH (9) followed by decrease in removal efficiency at higher pH values. This observation may be related to the hydrophobic interaction between the aromatic rings of dye molecules resulted from π–π stacking phenomenon, which make an agglomeration then precipitation of this dye at high acidic conditions^54,55^. However, the highest CV, MB and CR removal efficiencies were achieved at pH values of 5.5, 8.0 and 9.0, respectively. These pH values were fixed for all of the subsequent experiments for each dye.

#### Effect of sheet dose on the adsorption process

**Fig. 9:**
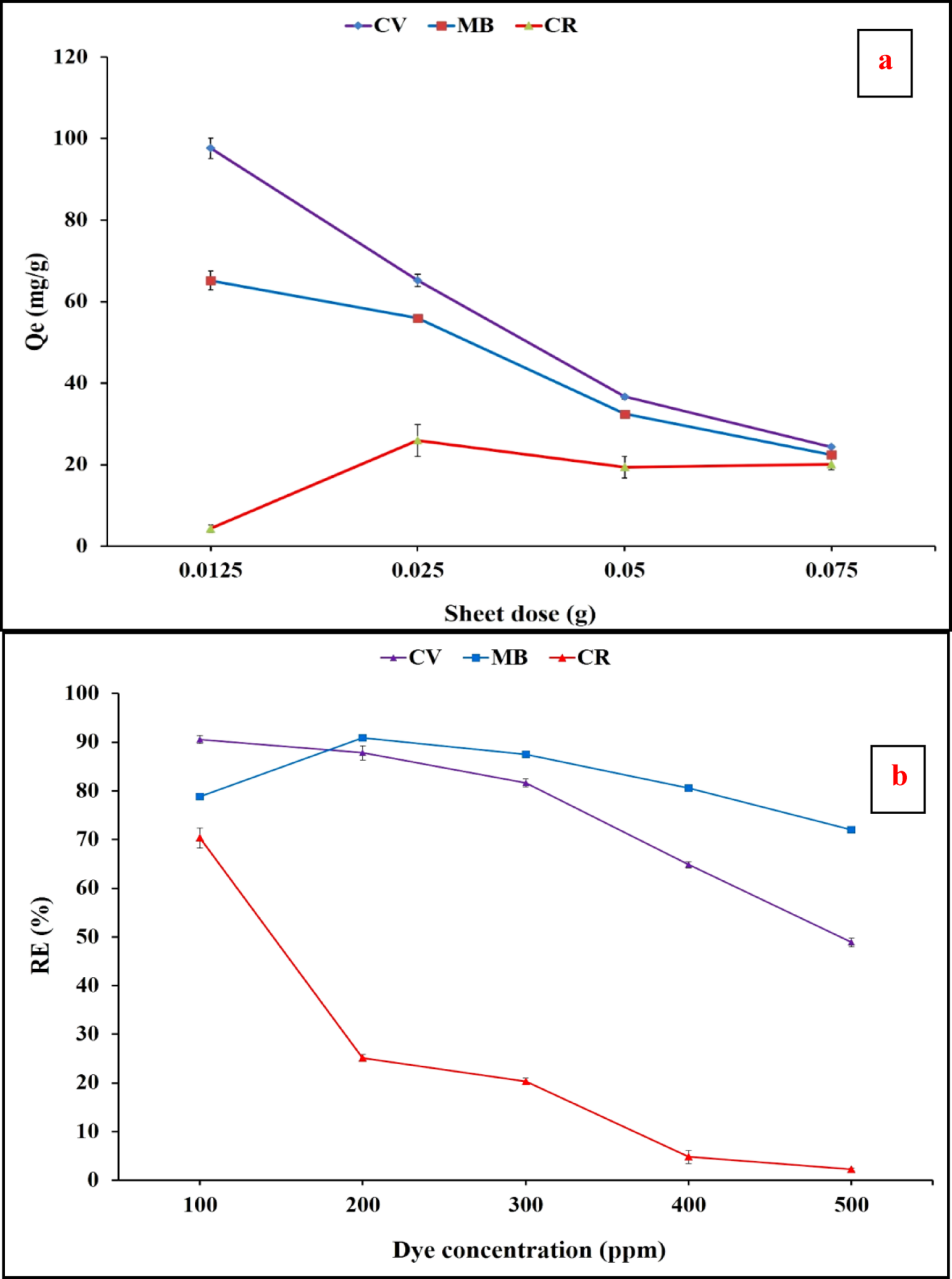

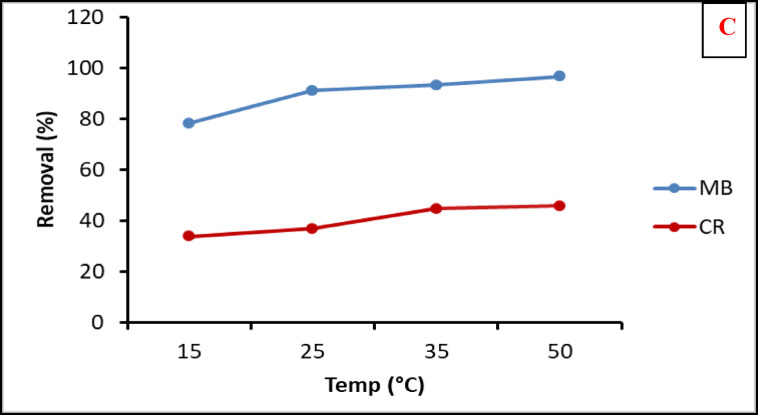
Effect of different sheet’s doses (**a**), initial dye concentration (**b**) and temperature (**c**) on removal efficiency of the tested dyes.

In the current study, different sheet doses of (0.0125, 0.025, 0.05 and 0.075 g) were investigated for CV, MB and CR removal. Figure 9a showed that 0.0125 g was the optimum sheet dose that achieved the highest crystal violet and methylene blue uptake with the maximum adsorption capacity of 97.62 and 65.19 mg/g, respectively By increasing the sheet doses, the pollutant concentration was decreased relatively to the number of adsorbent functional sites therefore, the adsorption capacity may decrease even the removal efficiency of dye increased. The removal efficiency was correlated to the concentration of dye, before and after adsorption, regardless of adsorbent dose. However, the adsorption capacity was related to the ability of adsorbent to adsorb pollutant molecules, regardless of pollutant concentrations^54^. Figure 9a displayed that; 0.025 g was the optimum sheet dose that achieved the highest CR uptake of 25.98 mg/g. This result may be attributed to the less affinity of this anionic dye towards the composite sheet`s functional-groups.

#### Effect of dyes concentration and temperature on the adsorption process

Different dye concentrations of CV, MB and CR ranging from 100 to 500 ppm were investigated using the previously optimized composite sheets. Figure 9b illustrated that the maximum removal efficiencies of 60.28, 53.22 and 45.03% for CV, MB and CR, at initial dye concentrations of 200, 200 and 100 ppm, respectively. After these concentrations, the removal efficiencies decreased which indicating a full saturation of the active adsorbent sits in the composite sheets. The adsorption of cationic dyes was largely higher than the anionic dye due to the nature of the reactive functional groups in algal biomass with anionic characters that have high affinity more towards cationic molecules. The higher adsorption of MB than CV may be affiliated to numerous intermolecular interactions (electrostatic or hydrophobic interactions) between the dye and the biosorbent surface^56^.

The effect of temperature on the adsorption of composite sheet towards MB and CR as represented dye for cationic and anionic dye, respectively, was indicated in Fig. 9-c. as the temperature increased the apparent adsorption of both dye was increased. This result may be due to by increasing the temperature the increased kinetic energy of dye and the more diffusion of these dye molecules into the interior pores of the polymer matrix which increase the change of attraction between the reactive functional groups of algal biomass and the dye molecules. The increased temperature may also break down the intra-hydrogen bonding algal molecules that will facilitate the attraction and formation of hydrogen bonding and electrostatic attraction between dye molecules and adsorbent.

### Adsorption isotherms

**Fig. 10:**
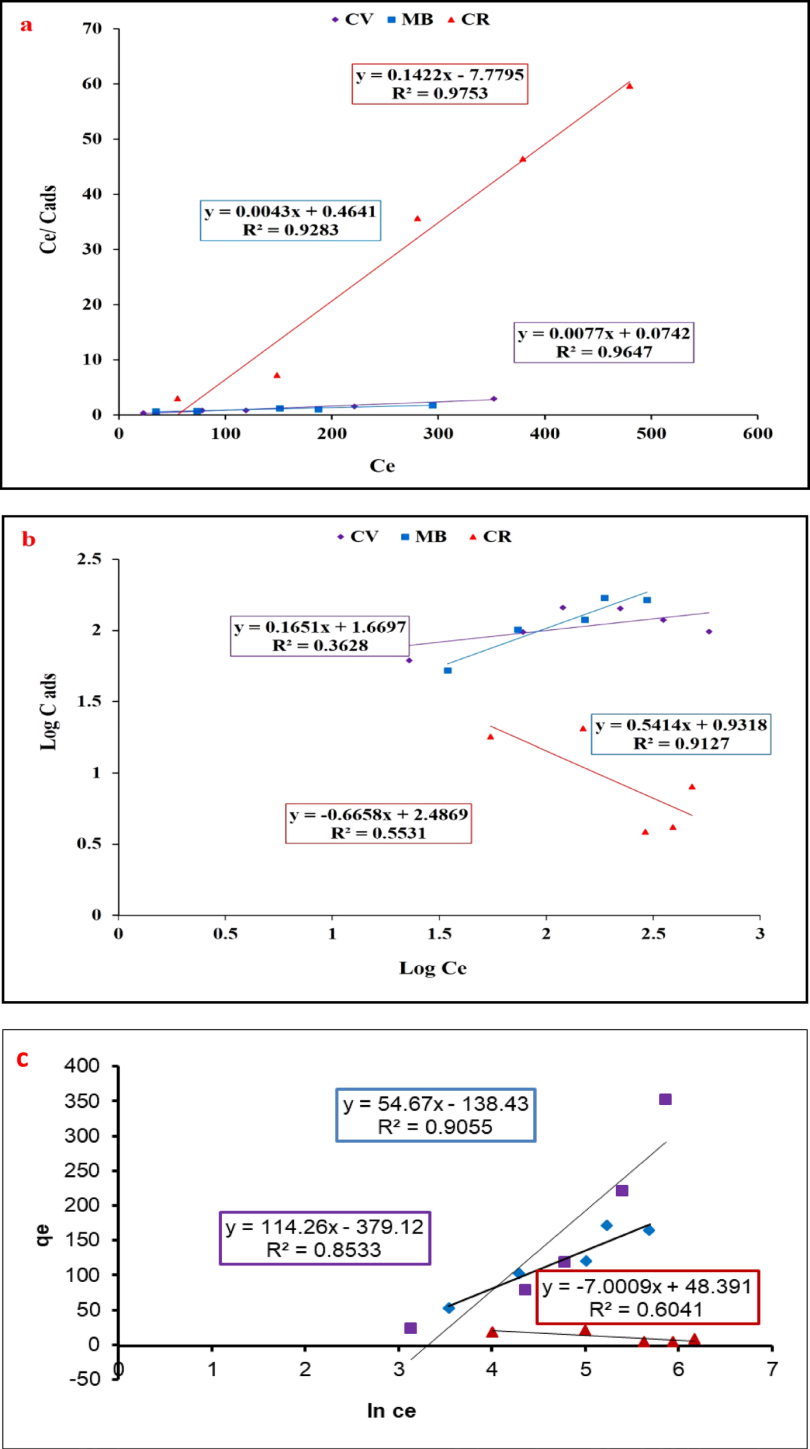

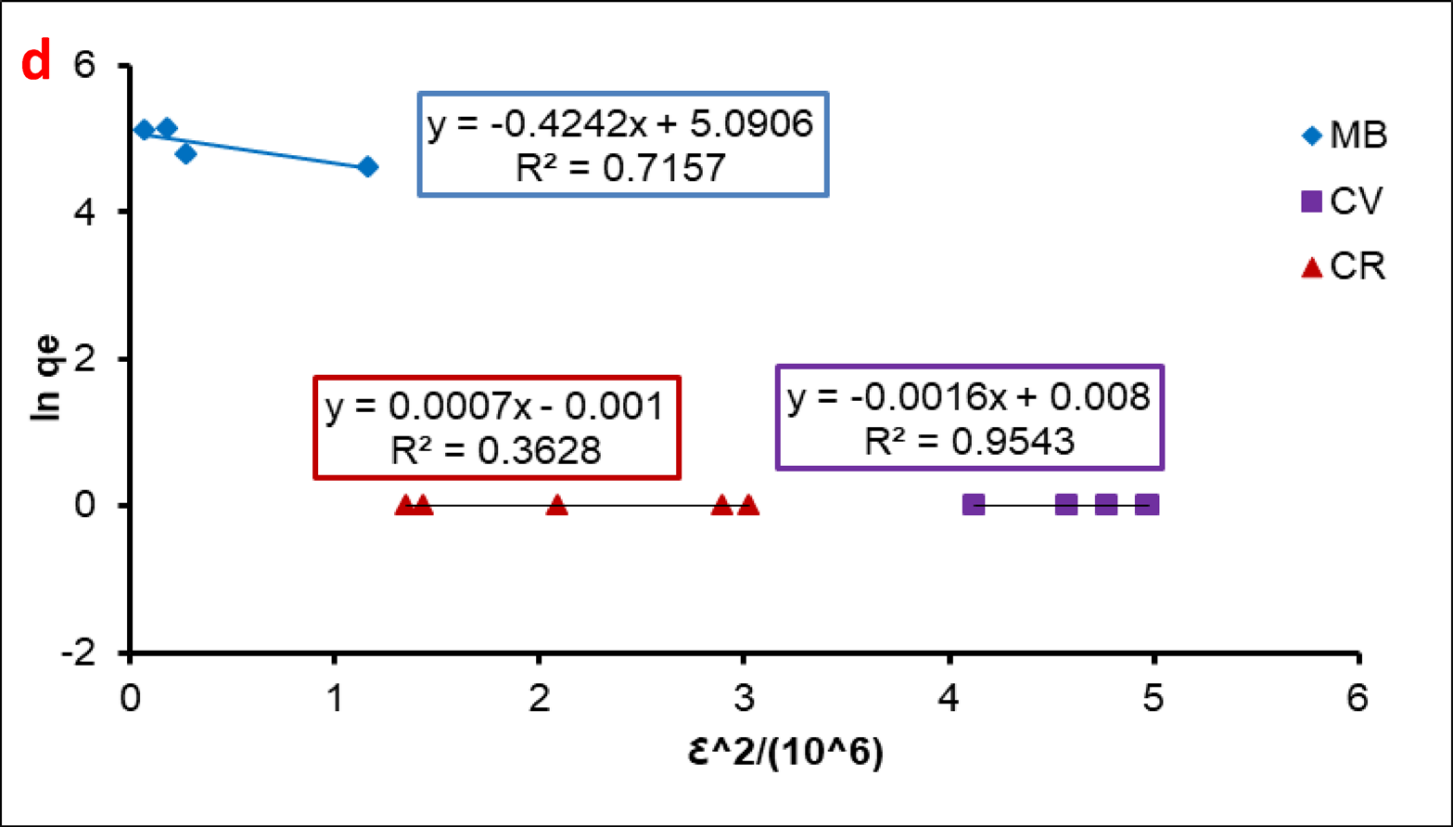
Adsorption isotherms of the composite sheets for the removal of CV, MB, CR; Langmuir (**a**), Freundlich (**b**), Temkin (**c**) and Dubinin-Radushkevich (**d**) isotherms.

Adsorption studies were analyzed using linear equations of Langmuir, Freundlich Temkin and Dubinin-Radushkevich adsorption isotherms. The Langmuir linear equation can be obtained by plotting of specific sorption (Ce/qe) and equilibrium concentration (Ce) of the dyes adsorbed by the composite sheets. While Freundlich isotherm is a linear plot between log Ce and Ce. The obtained results of Langmuir and Freundlich isotherms are presented in Fig. 10a, b and are summarized in Table 2. Temkin and Dubinin-Radushkevich adsorption isotherms are presented in Fig. 10c, d respectively. The adsorption data fitted better with the Langmuir model (see Table 2), with higher Regression factor (R^2^ values of 0.9753, 0.9283 and 0.9647 higher than that of other models. Following Langmuir isotherm indicates that the nature of the process of adsorption has a chemical nature (chemisorption) resulting in the formation of a monolayer of adsorbate on adsorbent active sites as previously reported by (Singh, et al., 204& Gupta, et al., 2008)^54,57^. The D-R isotherm model assumes that the adsorption takes place on the porous surface and evaluates the adsorption from an energetic point of view^33^. According to the D-R model, the adsorption potential varies according to the pore structure of the surface where the adsorption takes place. The E value gives information about adsorption mechanisms if it chemical or physical adsorption process. If the magnitude of the E value is between 8 and 16 kJ/mole, the chemical adsorption mechanism is in predominant, if it is smaller than 8.0 kJ/mole, the physical adsorption mechanism is the major^39^. The value of R^2^ is less than that of Langmuir and the value of E for the test dyes was less than 8 kJ/mole for MB and more than 16 for CV and CR which predict that the adsorption didn’t fit this adsorption model.

**Table 2:** Isotherm models parameters for the dye’s removal by the composite sheets.

Adsorption model	Isotherm parameters	MB	CV	CR
Langmuir	q_max_ (mg/g)	232.558	129.870	7.032
	b (L/mg)	0.0092	1.037	-0.018
	R^2^	0.9283	0.9647	0.975
Freundlich	N	1.8484	6.0606	-1.5037
	K_F_ (mg/g)	8.5310	46.665	306.196
	R^2^	0.9127	0.3628	0.5531
Temkin	a_t_ (l/g)	12.579	27.606	1004.36
	b_t_	46.08	22.05	359.83
	B (J/mole)	54.67	114.26	7.0009
	R^2^	0.9055	0.8533	0.6041
Dubinin-Radushkevich	Qs (mg/g)	162.487	1.008	1.001
	Kads (mol^2^/KJ^2^)	0.4242	0.0016	0.0007
	E (kJ mol^− 1^)	1.086	17.677	26.726
	R^2^	0.7175	0.9543	0.3628

### Kinetics studies

**Fig. 11:**
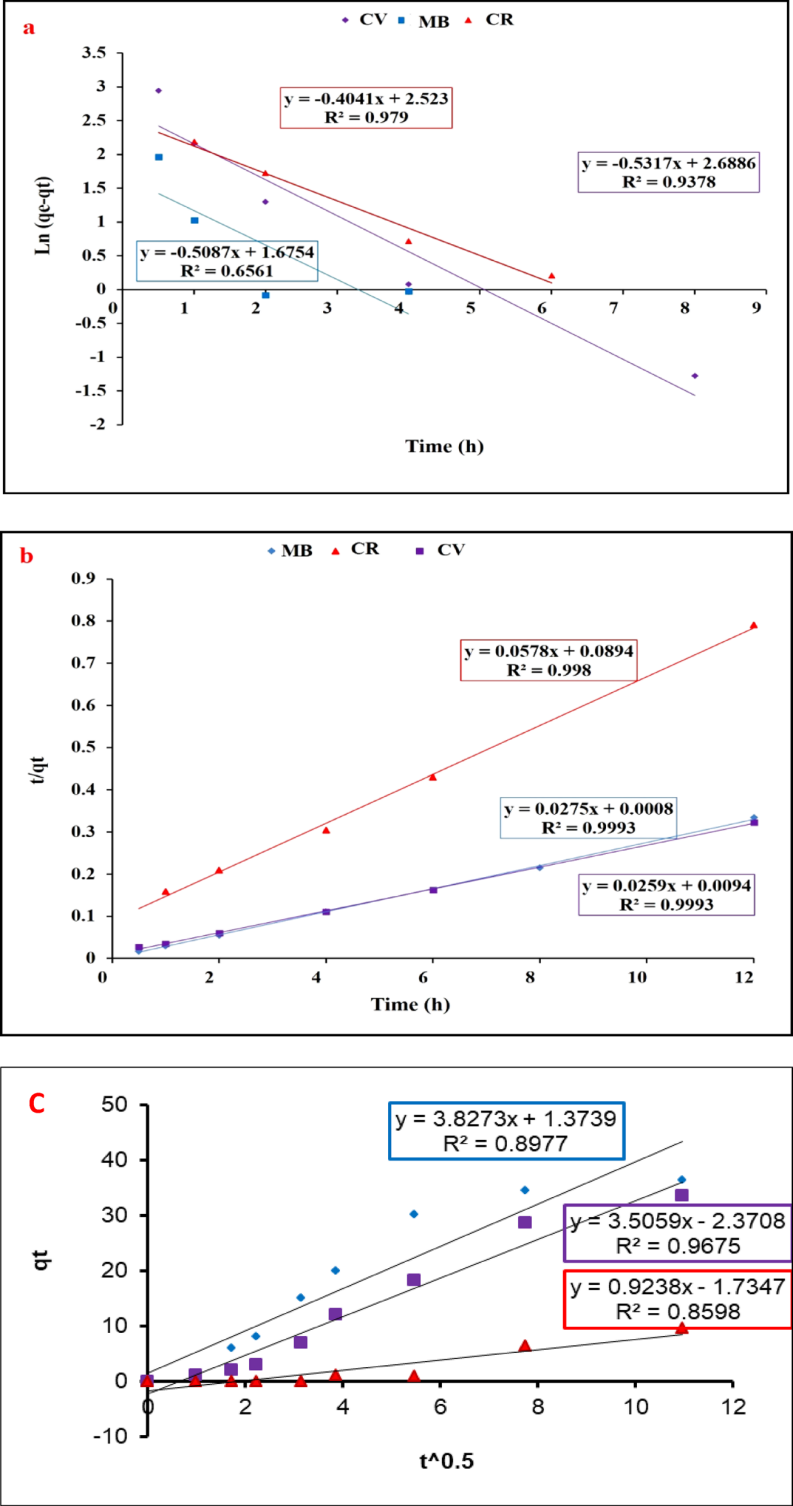
Adsorption kinetics of the composite sheets for the removal of CV, MB, CR; Pseudo-first order (**a**) Pseudo-second order (**b**). and Intraparticle diffusion model.

Equilibrium kinetics studies are important tools used to assess the affinity and capacity of the adsorption process. Pseudo-first, Pseudo-second-order and intraparticle diffusion kinetics are displayed in Fig. 11; Table 3. It was indicated that the adsorption kinetics matched better with the Pseudo-second-order model. The R^2^ values for all dyes were higher than 0.99, also the calculated (qe) values were closer the result of the experimental (qe) values (see Fig. 11; Table 3) which confirm that the biosorption followed a pseudo-second order model. For intraparticle diffusion model; the adsorption kinetics includes 3 mass transfer manners: the external diffusion: the transfer of adsorbate from the bulk of liquid into around the adsorbent; the internal diffusion (or intraparticle diffusion): the transfer of adsorbate into the pores of the adsorbent; and the adsorption onto the active site^35^. As shown in the figure and table; the straight lines of each dye didn’t pass through the origin and the regression factor R^2^ was less than 0.9 for two dyes and the recoded 0.9675 for crystal violet dye which indicate that CV was fit with this model however R^2^ is less that for the pseudo second order kinetics. This result can be confirmed that the second order is the most fitted model of kinetics for this adsorption.

These results indicated that the dyes biosorption could follow the chemisorption process. Wang and Chen stated that kinetics analysis of algal bio-sorbents was fit well with the pseudo-second order model and concluded that the chemisorption or the effective electrostatic interactions show essential role in biosorption^58^. Also, Kostic and his co-workers reported that, pseudo-second-order model may explain well the kinetics for dye biosorption onto Xanthated corn cobs^59^.

**Table 3:** Kinetic parameters of the Dye’s removal by the composite sheets.

	CV	MB	CR
*Pseudo-first order*			
q_e_ (mg/g) (calculated)	488.202	47.358	333.426
q_e_(experiment)(mg/g)	37.266	37.242	15.186
K_1_ (min^− 1^)	1.2245	1.171	0.9306
R^2^	0.9378	0.6561	0.979
*Pseudo-second order*			
q_e_ (mg/g) (calculated)	38.61	36.364	17.301
K_2_ (min^− 1^)	0.0714	0.9453	0.0374
R^2^	0.999	0.999	0.998
K_int_	3.8273	3.5059	0.9238
R^2^	0.8977	0.9675	0.8598

### Thermodynamics of adsorption

**Fig. 12:**
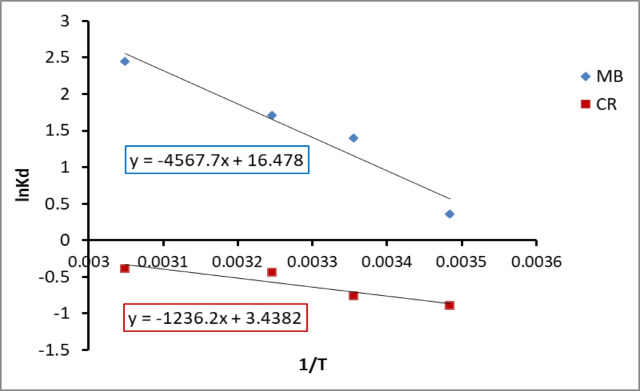
Adsorption thermodynamic (plot of lnKd vs. 1/T for MB and CR onto PES-S-ZnO composite sheet).

It can be observed from Fig. 12 and table that the values of ΔG were negative that indicated a spontaneous adsorption of methylene blue and crystal violet dye on the composite PES-S-ZnO sheet and the adsorption is highly favorable. The positive values of ΔH indicated that the adsorption process is endothermic nature and suggest chemical adsorption mechanism. The (ΔS) with positive values indicated the increased disorder and randomness at the solid liquid interface throughout the adsorption of organic dyes on the adsorbent sheet^60^ (Table 4).

**Table 4:** Thermodynamic parameters for the adsorption of MB onto PES-S-ZnO composite sheet.

Temperature (K)	ΔG^o^ (kJ/mol)	ΔH^o^ (kJ/mol)	ΔS^o^ (J/mol K)
287	– 859.13	37975.86	136.99
298	– 3463.42		
308	– 4375.21		
328	– 6668.67		
287	– 2132.75	10277.77	28.58
298	– 1888.26		
308	– 1128.3		
328	– 1060.62		

### Effect of mixed dye and cations on the adsorption of PES-S-ZnO composite sheet

**Fig. 13:**
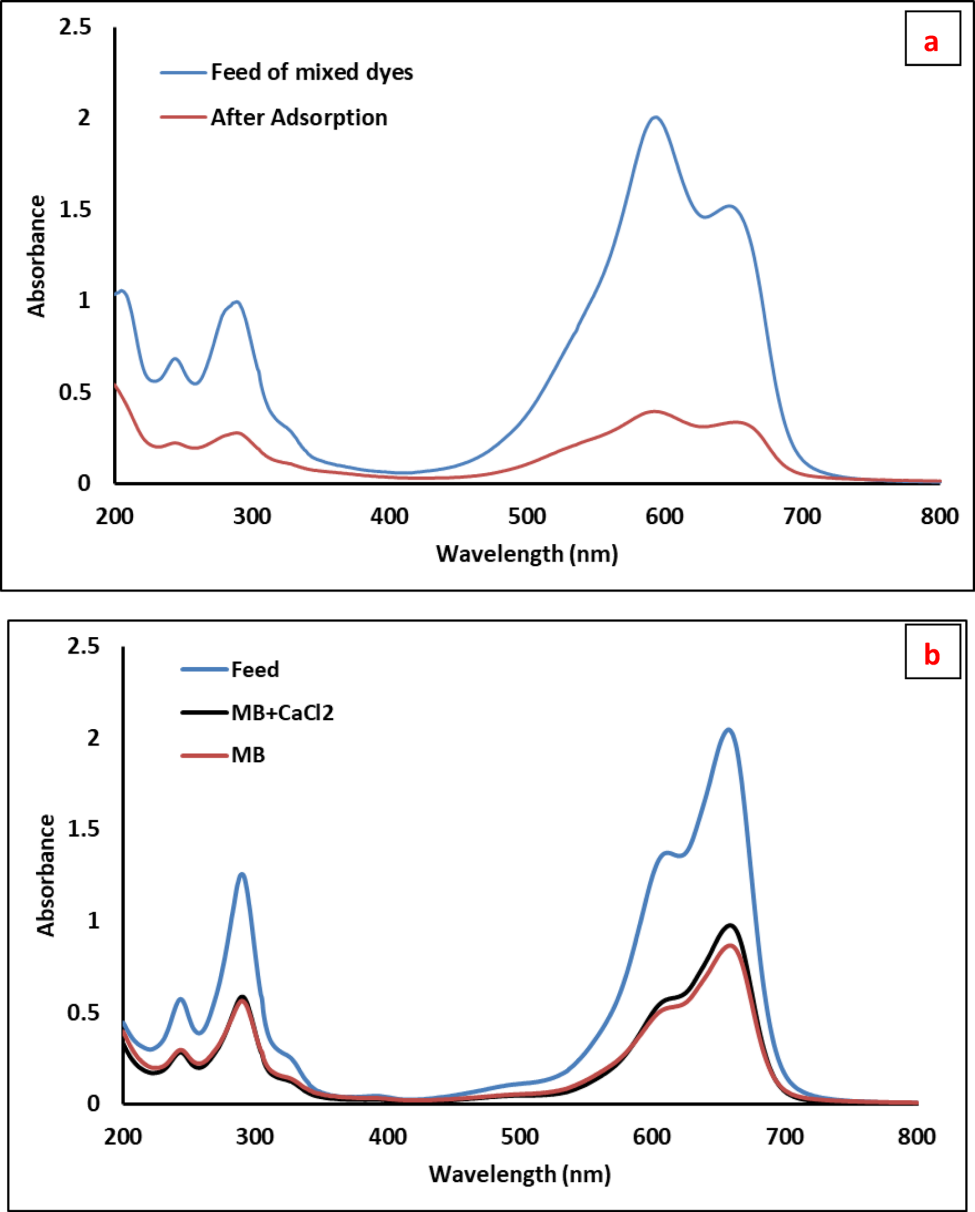
Uv-vis adsorption of mixed dyes before and after adsorption (**a**) and UV-Vis spectra of methylene blue before and after adsorption of composite sheet to the dye in presence and absence of CaCl_2_ (**b**).

The composite sheet composed of PES-S-ZnO was more selected to cationic dyes rather anionic dye. As shown in the optimization of adsorption, the composite has high affinity to cationic dyes; as a result we perform mixed dyes composed of cationic dyes (Methylene blue, Crystal violet and Nile blue in presence of sodium chloride). As shown in Fig. 13a; the UV-vis absorbance curve for (5 times diluted) mixed dye was appear with high intensity and after adsorption the intensity of all peaks of the mixed dye was greatly reduced and in consequence the concentration was greatly decreased due to removal of different dye simultaneously. The decrease in concentration of the crystal violet was slightly higher than the other dyes.

The effect of calcium cations on the adsorption of the composite to the organic dye was carried out by removal methylene blue dye in presence of calcium chloride salt. Methylene blue dye with concentration of 100 ppm was mixed with CaCL_2_ (100 ppm) and the adsorption was compared to the removal of only methylene blue. The result was displayed in Fig. 13b. The UV-vis adsorption curve was indicated that the presence of calcium chloride was slightly affect the adsorption of methylene blue dye due to the competition of calcium cations with cationic dye that let to decrease in the adsorption of organic dye.

### The mechanism of adsorption

**Fig. 14:**
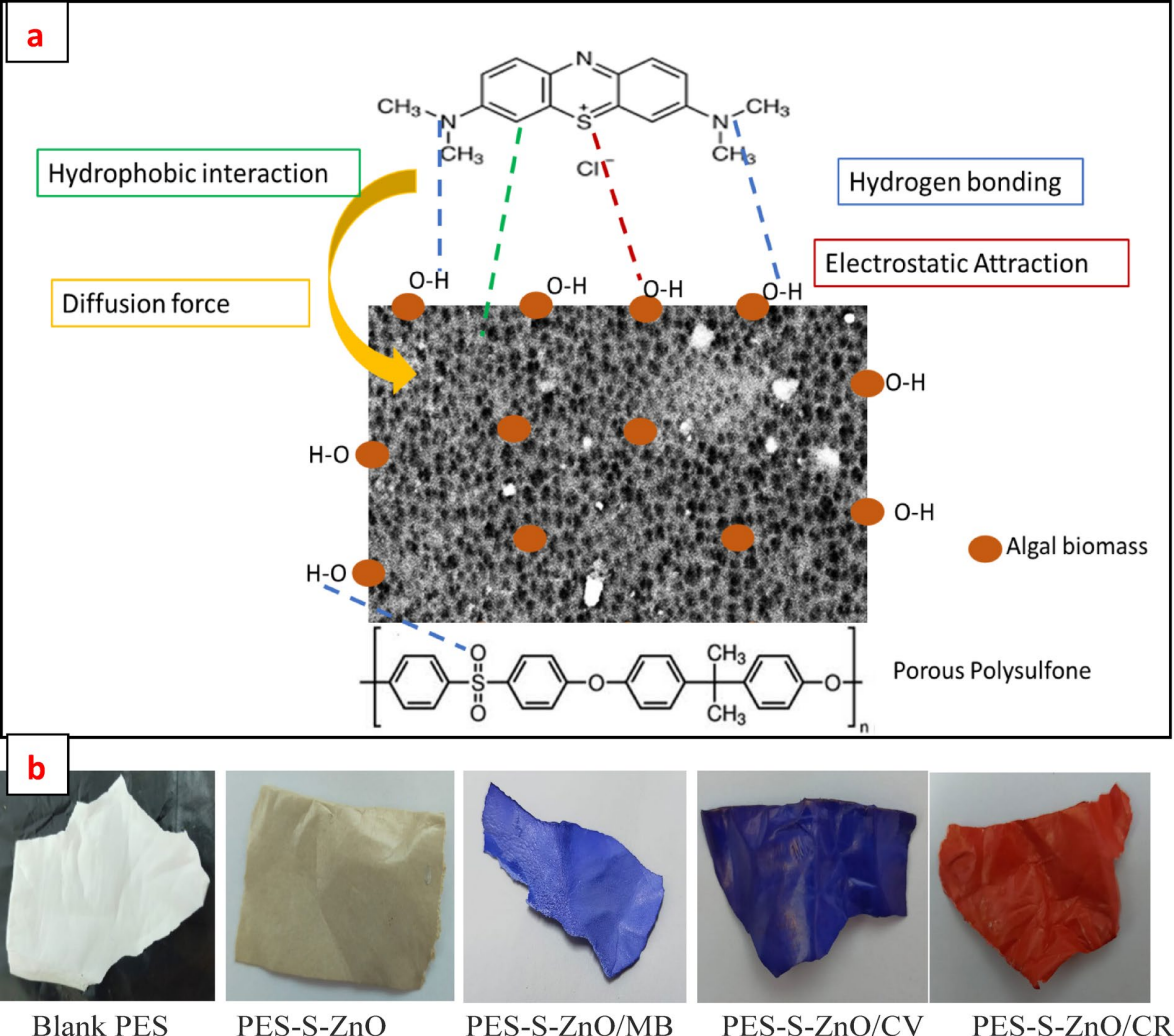
Proposed scheme for the mechanism of adsorption of PES-S-ZnO composite towards MB dye (**a**) and photo images for blank and composite sheet before and after adsorption of different dyes (**b**).

The adsorption of organic dyes onto PES-S-ZnO composite sheet involve different mechanism including hydrogen bonding between reactive functional groups on the cell wall of algal biomass and the dyes, also the electrostatic between the charged groups on dye molecule and polar groups on polymer and /or algal biomass^46^. Also the adsorption involve the hydrophobic interaction between the aromatic rings of dye molecules and aromatic groups of the polysulfone polymer via π–π stacking phenomenon and intra-diffusion of the dye molecule into the interior pores of the composite (Fig. 14a) in addition of weak vander waals attraction forces^49^. Figure 14b indicates images for blank and composite sheet before and after adsorption of different dyes that indicate the saturation of different dye onto composite adsorbent.

### Regeneration studies

**Fig. 15:**
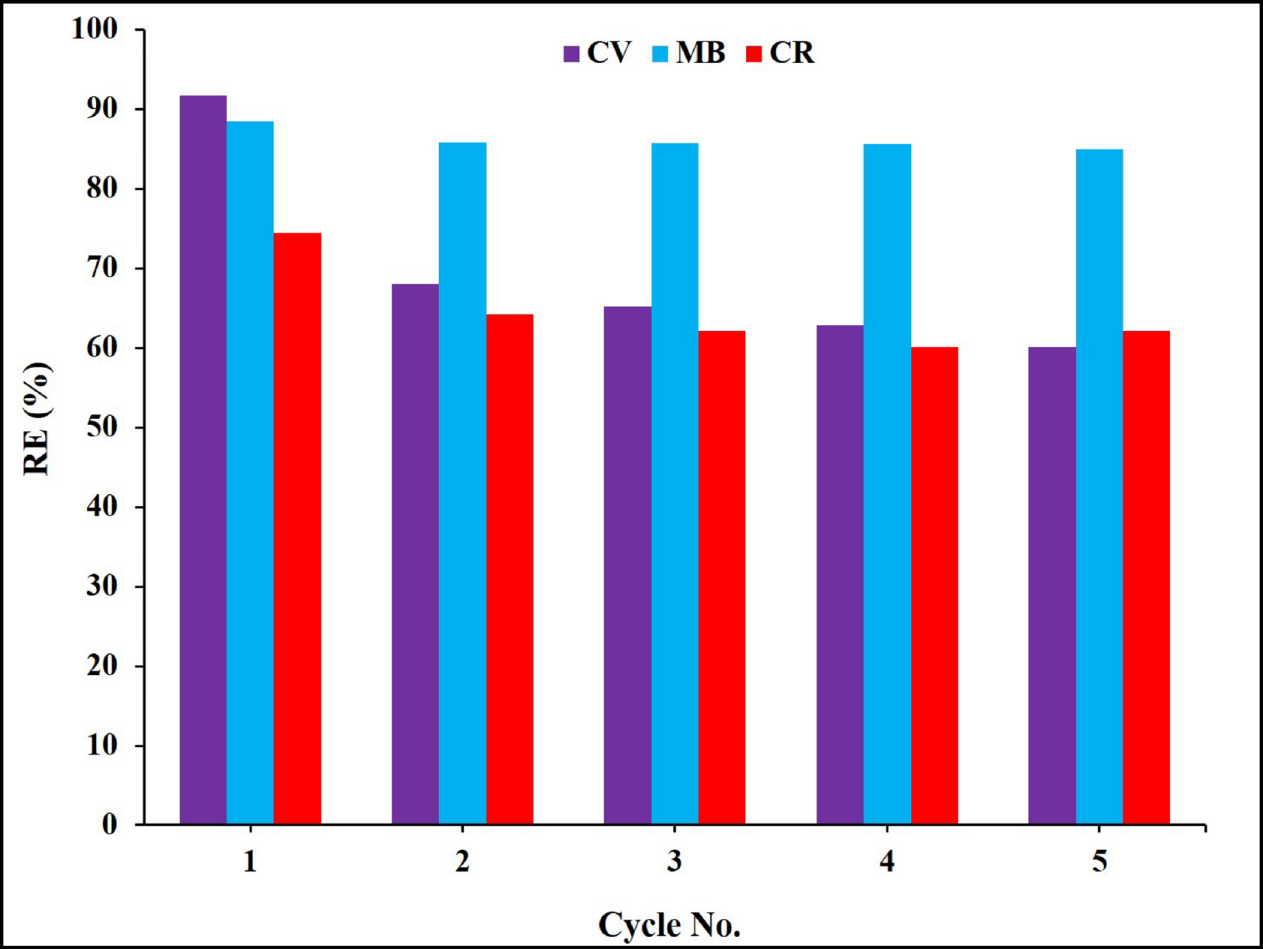
Regeneration study of composite sheets for many cycles’ removal, recovery and reuse of CV, MB and CR.

To evaluate the stability, usability and applicability of the composite sheets in the adsorption application, the sheets were applied in numerous adsorption and desorption cycles, as displayed in Fig. 15. After 5 regeneration cycles, the adsorption of the studied dyes followed a decline pattern in the order of 30% compared to fresh sheet. These results confirmed the efficiency of the composite sheets after several sorption desorption cycles, demonstrated their stability, usability and applicability for the adsorption process for several cycles^57^.

### Comparison with other adsorbents

To assess the effectiveness of the prepared algal polymer composites to remove organic dyes (cationic and anionic dye), the maximum adsorption capacity of PES-S-ZnO composites was compared with other adsorbents previously stated in the literature for treatment of MB, CV and CR dyes as shown in Table 5. One can see from the table that the maximum adsorption capacity values of the PES-S-ZnO composites prepared in this work was in line and even better than other adsorbents recorded in the literature used for the treatment of MB or CV or CR dyes. This confirmed that the prepared composite sheet is promising candidates for removal of cationic MB, CV and anionic dyes from aqueous solution with high efficiency.

**Table 5:** Comparison of maximum adsorption capacities for CV, MB and CR dye on different adsorbents.

Adsorbent	Dye	Adsorption capacity (mg/g)	References
Cellulose/activated carbon	MB	103.6	^61^
Starch/polyacrylic acid/ epichlorohydrin	MB	133.6	^62^
Chitosan/carboxy methyl cellulose/graphene oxide	MB	122.1	^63^
Modified rice husk	CV	12.6	^64^
Alginate / bentonite	CV	140	^65^
Chitosan / Talc / Cloisite 30B	CV	37.03	^66^
Zeolite/algae composite	CR	12.25	^67^
Fly ash	CR	22.12	^68^
PES-S-ZnO	MB	178.2	Current study
PES-S-ZnO	CV	150.5	Current study
PES-S-ZnO	CR	25.9	Current study

### Anti-fouling performance of the composite sheets

**Scheme 1:**
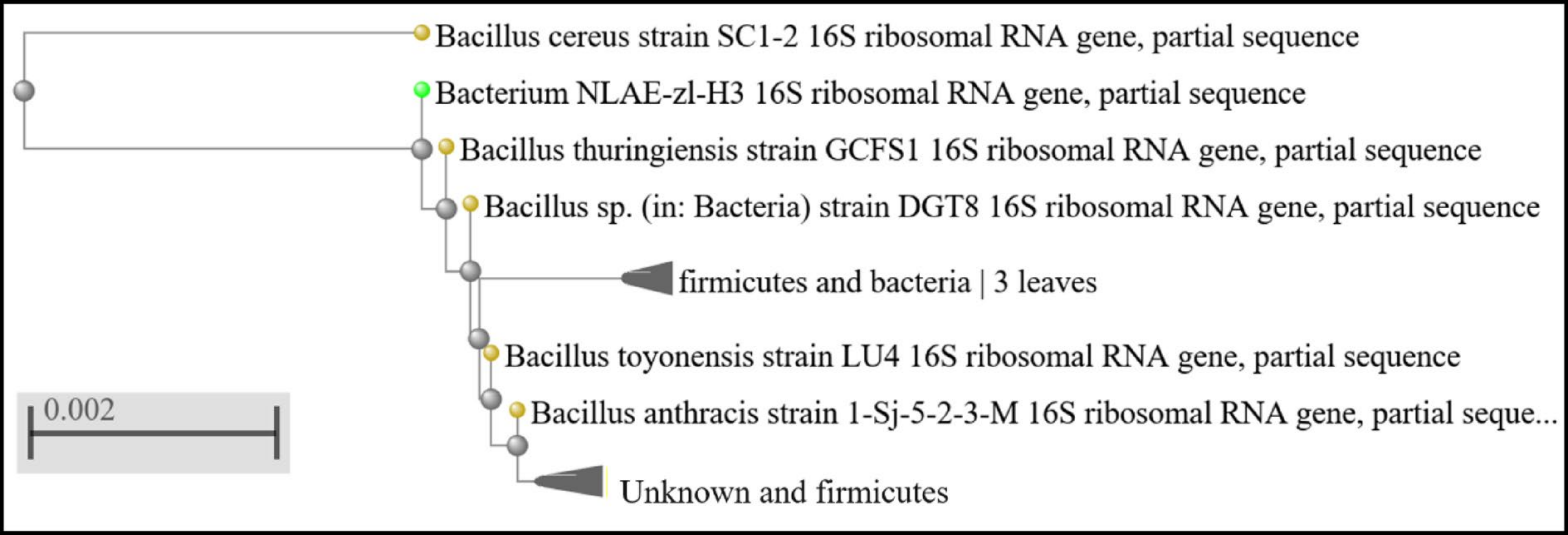
Neighbor-joining phylogenetic tree based on 16 S rRNA of the purified bacterial strain isolated from industrial wastewater sample and used for anti-biofouling test. Bootstrap values (1000 replicates). Scale bar represents 10% estimated sequence divergence.

**Fig. 16:**
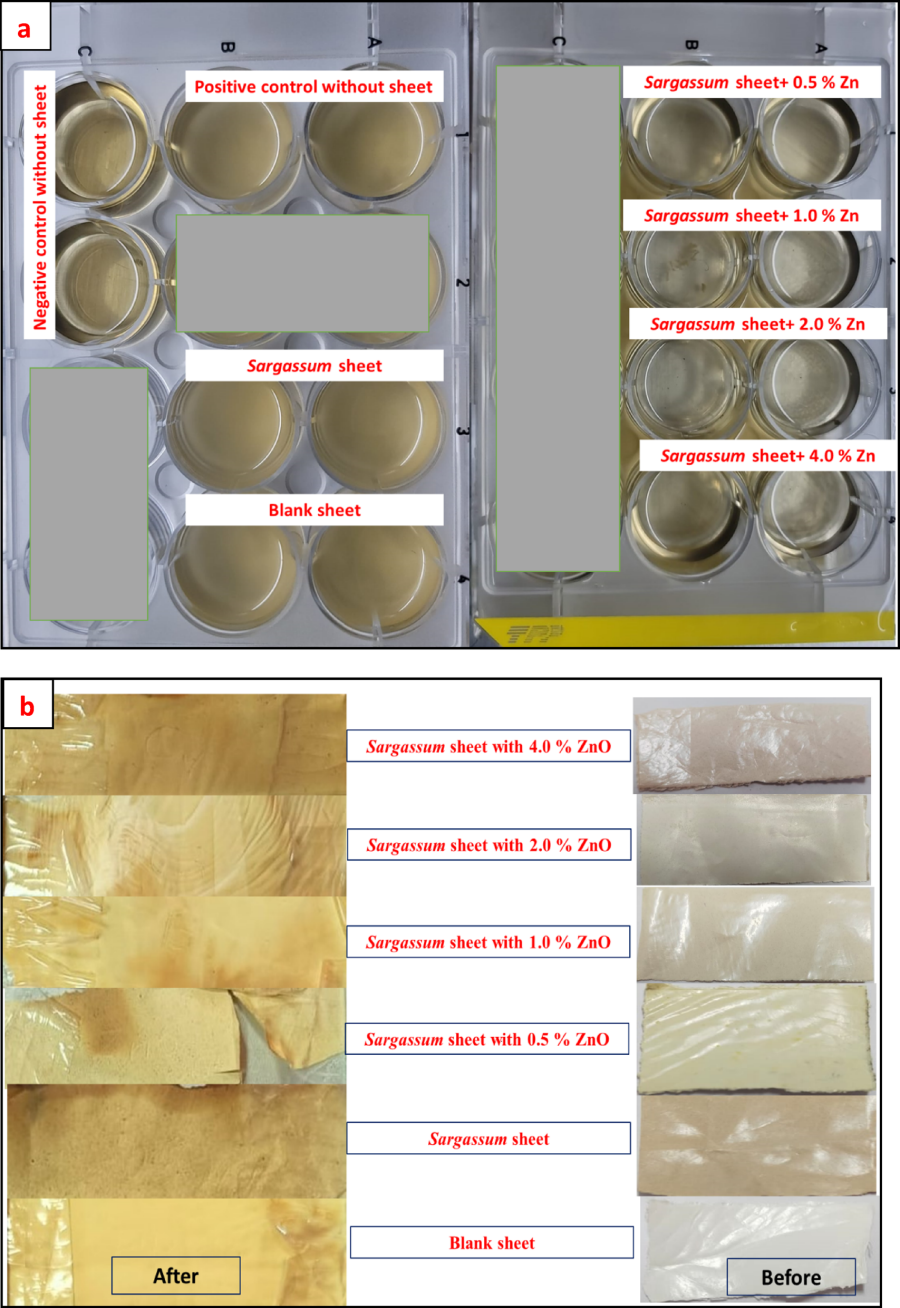
An image of antifouling test for the adsorbent sheets (**a**) and an image sheet before and after weathering test (**b**).

The results of anti-biofouling test of the composite sheets were shown in Fig. 16a; Table 6. The effect of addition of ZnO nanoparticles on the biofouling of the composite sheets was detecting by measuring the OD at 550 nm. Increase concentration of ZnO nanoparticles from 0.5 to 4.0% resulted in decrease in OD from 1.065 nm to 0.21 nm. This result was attributed mainly to the ZnO nanoparticle incorporated in the sheet matrix. The suggested antibacterial effect of the ZnO was related to two proposed mechanisms. The first one was related to the production of highly reactive oxygen species (ROS) upon excitation of ZnO in the presence of O_2_ and water, the ROS are produced, which in turn resulted in formation of O_2_^−^, OH^•^, and other oxygenated radical species^69^. The second mechanism was related to the toxicity effect of the Zn^2+^ ions that may be released or leached from the sheets^69^. According to Hinai et al., the number of colony-forming units (CFUs) of *E. coli* was significantly decreased for PES sheets modified with ZnO nanoparticles under a visible light irradiation, compared to that observed for the unmodified PES sheet^70^. The purified industrial strain, that was isolated, purified and applied in this study, was identified using 16 S rRNA. The results showed that the purified strain was *Bacillus cereus* with query coverage of 98% and an identity of 96% (Scheme 1).

**Table 6:** The anti-biofouling test of the composite sheets in comparison to positive control and blank sheet.

Sheet’s name	OD of 550 nm ± standard deviation
Positive control	1.36 ± 0.06
Blank sheet (PES)	1.275 ± 0.075
PES-S	1.16 ± 0.14
PES-S-ZnO (0.5%)	1.065 ± 0.145
PES-S-ZnO (1.0%)	0.71 ± 0.03
PES-S-ZnO (2.0%)	0.47 ± 0.06
PES-S-ZnO (4.0%)	0.21 ± 0.03

### Accelerated weathering test of the composite sheets

The results of weathering test of the composite sheets indicated that the brownish color intensity (high probability of degradation) was increased in the composite sheets in comparison to PES sheet (Fig. 16b). The result displayed that, by incorporation of algal biomass within the polymer sheet, the probability for degradation of the polymer sheet increased. Furthermore, by increasing in the ZnO concentrations in the algal polymer sheets leads to an increasing in the composite sheet’s degradation^71^. These results indicated that the formulated composite sheets were a promising candidate for industrial wastewater treatments with biodegradation properties that decrease the environmental problem after the end life time of the sheet functionality.

## Conclusion

Preparation of polyethersulfone sheet impregnated with *Sargassum dentifolium* and ZnO nanoparticles was successfully performed for removing of different organic dyes; crystal violet (CV), methylene blue (MB), and Congo Red (CR) dyes from wastewater. The prepared composite adsorbent was evaluated and confirmed using different analytical techniques. The composite sheet showed enhanced adsorption capability towards cationic dyes rather than anionic one. Adsorption isotherms displayed good represented data with Langmuir model for the composite sheets and the kinetic data suggested that the adsorption matched with pseudo-second order model. The used sheet can be recycled for five cycles without losing of apparent efficiency by using ethanol. The presence of ZnO-NP in the polymer- algal sheets leads to an increasing in anti-biofouling and enhancing degradation performance of the sheets. The low cost material and reliable technique facilitate the fabrication of biodegradable and antifouling adsorbent composite sheet.

## Data Availability

The datasets used and/or analyzed during the current study available from the corresponding author on reasonable request.
